# Genetic Insights into Pediatric Polycystic Kidney Disease and Overlapping Ciliopathies: A Narrative Review

**DOI:** 10.3390/genes17101252

**Published:** 2026-10-10

**Authors:** Valeria Chirico, Filippo Tripodi, Fabiola Cassaro, Antonio Lacquaniti, Salvatore Arena, Carmelo Romeo, Roberto Chimenz

**Affiliations:** 1 Pediatric Nephrology and Dialysis Unit, Department of Human Pathology of Adult and Childhood “Gaeta-no Barresi”, University of Messina, 98124 Messina, Italy; 2 Department of Biomedical and Dental Sciences and Morpho-functional Imaging, University of Messina, 98124 Messina, Italy; fabiola.cassaro@studenti.unime.it; 3 Nephrology and Dialysis Unit, Department of Internal Medicine, Papardo Hospital, 98158 Messina, Italy; 4 Unit of Pediatric Surgery, Department of Human Pathology of Adult and Childhood “Gaetano Barresi”, University of Messina, 98124 Messina, Italyromeoc@unime.it (C.R.)

**Keywords:** autosomal dominant polycystic kidney disease, ADPKD, ARPKD, genotype–phenotype correlation, ciliopathies, next-generation sequencing, NGS, precision nephrology, genetic testing, pediatric polycystic kidney disease

## Abstract

Pediatric cystic kidney diseases lie at the intersection of autosomal dominant polycystic kidney disease (ADPKD) and a broad spectrum of ciliopathies. The pediatric phenotype often reflects a spectrum that includes ARPKD-like presentations, nephronophthisis-related disease, Joubert syndrome-associated kidney involvement, Bardet–Biedl syndrome, and other ciliopathies that can mimic or overlap with PKD. While ADPKD is traditionally attributed to pathogenic variants in *PKD1* and *PKD2*, the widespread deployment of next-generation sequencing has uncovered causal variants in novel loci, including *GANAB* (~0.3%), *DNAJB11* (~0.1%), *ALG5*, *ALG9*, and recently, *IFT140* and *NEK8*. This review outlines the expanding genetic and clinical landscape of ADPKD, integrating recent cohort data and molecular insights. We discuss radiologically atypical presentations, overlapping syndromic ciliopathies, and the mechanisms of metabolic reprogramming and primary ciliary dysregulation in cyst pathogenesis. Recent milestones in clinical trials and emerging therapeutic strategies, including perspectives on early risk stratification before the transition to adulthood, are also evaluated. Ultimately, this review emphasizes the necessity of gene-based classifications and precise genotype–phenotype correlations, aligned with the 2025 KDIGO Clinical Practice Guideline for ADPKD, to optimize risk stratification and advance precision medicine in the clinical management of ADPKD.

## 1. Introduction

Autosomal dominant polycystic kidney disease (ADPKD) is the most common inherited renal disorder and the leading genetic cause of chronic kidney disease (CKD) in adulthood.

Based on comprehensive European registry and population sequencing data updated between 2017 and 2018, the clinical prevalence of ADPKD is estimated to range between 1 in 1100 and 1 in 2500 individuals [1,2,3,4].

The disease is characterized by the progressive development and bilateral expansion of fluid-filled renal cysts, which ultimately lead to end-stage kidney disease (ESKD) in the majority of patients.

Affected individuals exhibit a significantly accelerated decline in estimated glomerular filtration rate (eGFR) compared to the general population, averaging a loss of 4–6 mL/min/1.73 m^2^ per year, in contrast to the physiological decline of approximately 1 mL/min/1.73 m^2^ per year observed with normal aging.

While ADPKD typically presents during the third or fourth decade of life due to a progressive increase in total kidney volume (TKV), its phenotypic spectrum is remarkably broad. Severe cases can become overt during childhood or even in the prenatal period, with pediatric-onset disease accounting for approximately 2–5% of all diagnosed cases. Among these pediatric cohorts, nearly 3% of pathogenic variant carriers experience either early-onset or unusually aggressive disease course [5,6,7]. In children, the early development of renal cysts may reflect altered embryonic kidney development in utero, presenting as a manifestation of a multicystic dysplastic kidney or a broader ciliopathy [7].

Early diagnosis carries profound prognostic implications; while many pediatric cases are incidentally detected via renal ultrasound (US) during evaluations for abdominal pain, trauma, or through parental screening, individual genotype–phenotype correlations remain the primary determinant of long-term prognosis. Beyond its primary renal manifestations, ADPKD is a systemic disorder resulting from the ubiquitous expression of polycystins across various extrarenal tissues [8,9]. The condition encompasses a spectrum of dominantly inherited conditions characterized by renal cystic disease and multi-organ extrarenal involvement linked to a specific pathogenic gene variant [10]. However, for individuals presenting with an ADPKD-spectrum phenotype who either lack genetic testing, display no identifiable pathogenic variants, or carry variants of uncertain significance (VUS) in minor modifier genes (such as *ALG6, ALG8, or PKHD1*), the clinical designation of ADPKD remains standard [10,11].

This review analyzes the expanding genetic and clinical spectrum of ADPKD by synthesizing recent breakthroughs in molecular diagnostics, imaging-based classifications, and the mechanisms of phenotypic variability across the lifespan.

We highlight novel disease-associated genes, atypical and early-onset presentations, and the emerging role of precision medicine in refining prognosis and structuring personalized therapeutic interventions.

Integrating recent cohort data and international consensus statements, this work establishes an updated clinical paradigm to optimize risk stratification and long-term ADPKD management.

### Method Section

A computerized literature search was performed across PubMed, Scopus, and Google Scholar databases. The time period covered by the reviewed literature spans from database inception up to 2026, incorporating the most recent molecular and clinical updates. The search was performed using combinations of the following keywords: “pediatric polycystic kidney disease”, “ADPKD”, “ARPKD”, “ciliopathies”, “next-generation sequencing”, “genetic testing”, and “genotype-phenotype correlation”, “pediatric polycystic kidney disease”. Only peer-reviewed articles, clinical trials, consensus guidelines, and reviews published in the English language were considered. The reference lists of the retrieved articles were also manually screened to identify additional relevant studies. Literature selection was strictly focused on pathophysiological, genetic, and clinical data with direct relevance to pediatric manifestations, early risk stratification, and ciliary development, while global adult-centric management data were omitted to maintain a dedicated pediatric scope.

## 2. Genetic Basis of ADPKD

Pathogenic variants in *PKD1* and *PKD2* account for approximately 85% and 15% of typical clinical cases, respectively. However, these two main loci do not fully explain the broad clinical variability encountered in clinical practice.

Recent evidence has identified additional causal genes, such as *GANAB*, *DNAJB11*, and members of the ALG gene family, which are typically linked to milder, late-onset, or radiologically atypical phenotypes [2,12,13].

Aligning with these discoveries, the Kidney Disease: Improving Global Outcomes (KDIGO) 2025 Clinical Practice Guidelines emphasize a gene-based nomenclature system (e.g., *ADPKD-PKD1, ADPKD-PKD2, ADPKD-IFT140)*.

This precise genetic classification refines both diagnostic accuracy and prognostic stratification, informing target-driven therapeutic interventions [10].

### 2.1. The Complexity of the PKD1 and PKD2 Loci

The *PKD1* gene is highly complex, spanning approximately 53 kb of genomic DNA. A major challenge in its molecular analysis is that the 5′ genomic region consists of highly homologous duplicated sequences (pseudogenes), sharing nearly 98% sequence identity with the master gene [14,15].

The presence of these six pseudogenes complicates standard genetic sequencing, frequently leading to false negatives or misalignments, which necessitates specialized approaches such as locus-specific long-range polymerase chain reaction (LR-PCR) amplification to selectively amplify the authentic PKD1 locus [14,15]. Relying solely on standard molecular diagnostics or conventional whole-exome sequencing (WES) significantly limits the sensitivity and specificity required to identify pathogenic variants between exons 1 and 33 [16,17,18].

Accordingly, extended genetic panels and tailored bioinformatic pipelines are increasingly required to ensure an accurate molecular diagnosis [16].

To date, more than 1200 pathogenic *PKD1* variants have been reported; most of these are private (family-specific), and no true mutational hotspots exist [17,18]. Furthermore, approximately 10–15% of patients present without a family history of the disease, indicating that de novo pathogenetic variants occur at a considerable rate [19,20,21]. The ongoing identification of novel in-frame deletions, somatic mosaicism, and local “second-hit” mechanisms further expands the mutational spectrum, driving significant phenotypic variability and compounding current diagnostic challenges [19,22,23,24,25,26]. The identification of biallelic hypomorphic variants in the *PKD1* gene can result in in utero onset, challenging the conventional boundaries between autosomal dominant and autosomal recessive inheritance patterns. While completely penetrant biallelic pathogenetic variants are typically fatal or embryonically lethal, combinations of weaker alleles can lead to Very-Early-Onset (VEO) ADPKD. Clinically, truncating pathogenetic variants in *PKD1* generally precipitate severe forms of the disease, and variations located in the 5′ region are linked to an earlier onset of ESKD compared to those residing in the 3′ region [27,28,29,30,31,32]. At the structural level, *PKD2* encodes polycystin-2, a calcium-permeable channel belonging to the transient receptor potential (TRP) family [12,14,17,22,32,33,34,35]. Pathogenic variants in *PKD2* contribute to approximately 15% of cases, with around 250 identified variants, half of which are distinct. While pathogenetic variants in *PKD1* and *PKD2* cause similar structural downstream changes, *PKD1* variants usually result in earlier cystogenesis, a greater number of cysts, and accelerated progression to ESKD [12,14,17,22,32,33,34,35].

### 2.2. Genomic Rearrangements and the TSC2/PKD1 Contiguous Gene Syndrome

Multiplex Ligation-dependent Probe Amplification (MLPA) is standardly utilized to detect copy number variations (CNVs) across specific, predefined genomic regions associated with cystic disorders [16,25,36].

Large deletions impacting both *TSC2* and *PKD1*, which lie adjacently on chromosome 16p13.3, lead to the *TSC2/PKD1* contiguous gene syndrome (CGS) upon concurrent inactivation of both loci [37,38]. This syndrome is frequently associated with early-onset (EO) ADPKD presentations and classic tuberous sclerosis features, precipitating an accelerated loss of kidney function [39].

Conversely, uncommon non-contiguous deletions provide further insight into intricate genomic recombination processes [37,38,40,41]. Renal cysts in patients with *TSC2/PKD1* CGS exhibit a distinct morphology, characterized by severe bilateral overgrowth, marked kidney enlargement, and a high propensity for rapid progression. This presentation differentiates CGS from classic tuberous sclerosis complex (TSC), in which renal cysts are usually smaller and fewer. At the molecular level, *TSC* pathogenetic variants disrupt the mechanistic target of rapamycin (mTOR) pathway, resulting in constitutive activation that drives accelerated cyst growth [38,40,41]. Complex ADPKD phenotypes, including VEO disease and CGS, therefore require multigene panel sequencing to evaluate all potentially involved cystic kidney disease loci.

### 2.3. The Expanding Spectrum of Minor Loci and ER-Associated Genes

Large-scale cohort studies have established monoallelic loss-of-function variants in *IFT140*, a gene historically associated only with recessive ciliopathies, as key drivers of an atypical ADPKD phenotype characterized by large, sparse cysts and relatively preserved kidney function [42,43,44]. *IFT140* is currently recognized as the third most frequent cause of ADPKD-spectrum disease.

In contrast, biallelic *IFT140* variants cause severe syndromic ciliopathies, such as Jeune asphyxiating thoracic dystrophy and Mainzer–Saldino syndrome [42,43,44,45,46,47], whereas alternative homozygous configurations present as isolated Leber congenital amaurosis or retinitis pigmentosa [48].

Other genes involved in atypical or overlapping traits include *LRP5, COL4A1–A5*, and endoplasmic reticulum (ER)-associated loci such as *ALG8, ALG9, GANAB*, and *DNAJB11*.

Specifically, monoallelic loss-of-function variants in *ALG8* have been established as causal factors in the atypical polycystic spectrum, presenting with multiple hepatic and renal cysts typically characterized by a mild course and preserved kidney function. This monoallelic presentation clinically overlaps with autosomal dominant polycystic liver disease (ADPLD) phenocopies, whereas biallelic pathogenetic variants in *ALG8* lead to a severe, systemic autosomal recessive congenital disorder of glycosylation (*ALG8*-CDG) [10,11,49,50].

Pathogenetic variants in type IV collagen genes (*COL4A3–A5*) can produce bilateral renal cysts even in the absence of classic Alport syndrome features, and dual-mutation carriers of *COL4A5* variants have demonstrated digenic or synergistic inheritance patterns [51,52,53]. Furthermore, ER-associated genes regulate atypical cystogenesis through their impact on protein N-glycosylation and the ciliary trafficking of the polycystin complex, accounting for distinct disease patterns in selected cohorts [10,11,49,50].

Additionally, the serine/threonine kinase *NEK8* has recently been established as a causal gene for ADPKD. Monoallelic *NEK8* pathogenetic variants present with enlarged kidneys and limited extrarenal manifestations, yet drive rapid disease progression. While these monoallelic variants are linked to progressive VEO-ADPKD, biallelic pathogenic variants in *NEK8* remain strictly linked to severe, syndromic ciliopathies (renal-hepatic-pancreatic dysplasia) [54,55,56,57].

Given this extensive genetic heterogeneity and clinical overlap with alternative ciliopathies, comprehensive molecular testing is essential. Gene panels using next-generation sequencing (NGS) represent a standard molecular technique, allowing simultaneous analysis of a large number of candidate loci [56,57]. However, targeted panels may miss newly identified loci, and standard whole-exome sequencing (WES) frequently lacks the specialized coverage and read depth required to distinguish the authentic *PKD1* master gene from its six highly homologous pseudogenes. Consequently, whole-genome sequencing (WGS) has increasingly become the preferred diagnostic choice, as recent cost reductions allow clinicians to utilize its superior alignment precision to reliably detect complex structural and causal variants across the lifespan [55,56,57,58,59,60,61,62,63,64].

Approximately 10% of clinically diagnosed ADPKD cases lack identifiable *PKD1* or *PKD2* variants, reflecting deep intronic pathogenetic variants or the involvement of alternative cystic loci.

Consequently, contemporary multigene diagnostic panels for polycystic and cystic kidney diseases should not be restricted to *PKD1* and *PKD2*; they should concurrently integrate *IFT140, GANAB, ALG8, ALG9, HNF1B*, and *DNAJB1*1, alongside alternative disease phenocopies and ciliopathy-associated genes such as *PKHD1, DZIP1L, FLCN,* and the *NPHP* and *BBS* family loci [58,59].

### 2.4. Overlapping Phenotypes: HNF1B-Related Disease and ADPKD

Finally, pathogenetic variants in genes linked to hereditary tubulointerstitial diseases (HTD) may lead to cystic kidney development. These hybrid phenotypes, which exhibit both small renal cysts and interstitial fibrosis, underscore the clinical necessity of distinguishing ADPKD from autosomal dominant tubulointerstitial kidney disease (ADTKD) and related progressive nephropathies [65,66,67]. An accurate differential diagnosis is essential to separate these specific conditions. While typical ADTKD presenting with normal or small-sized kidneys involves progressive tubulointerstitial fibrosis without significant kidney enlargement, *HNF1B*-associated disease represents a distinct developmental form (ADTKD-*HNF1B*) characterized by cortical cysts, renal dysplasia, MODY5 diabetes, and pancreatic hypoplasia. In contrast, *DNAJB11* pathogenetic variants produce a unique hybrid clinical picture: structurally, the primary defect leads to tubulointerstitial fibrosis, but clinically it closely mimics an atypical, late-onset ADPKD with multiple bilateral renal and liver cysts despite a preserved total kidney volume. Pathogenetic variants in the hepatocyte nuclear factor 1-beta gene (*HNF1B*) cause ADTKD-*HNF1B*, a disorder characterized by a highly variable clinical phenotype due to structural or sequence alterations in the 17q12 genomic region. The disorder shows incomplete penetrance, and approximately 50% of cases arise from de novo pathogenetic variants. *HNF1B* encodes a 557-amino-acid transcription factor essential for normal renal, pancreatic, and gastrointestinal development; it regulates the expression of several *PKD*-related genes, including *PKD2* and *PKHD1*. Pathogenic variants in *HNF1B* encompass full gene deletions, which usually co-occur with neighboring gene deletions within the 17q12 microdeletion syndrome, as well as intragenic missense and truncating *HNF1B* loss-of-function variants. Regarding genotype–phenotype correlations, neurodevelopmental and neuropsychiatric manifestations are strongly associated with large full gene deletions that include neighboring loci in the 17q12 region, whereas missense and truncating loss-of-function variants are typically linked to progressive isolated CKD and maturity-onset diabetes of the young type 5 (MODY5).

Recent cohort analyses demonstrate that neurodevelopmental phenotypes, including autism spectrum disorder and learning difficulties, can also occur in individuals carrying isolated intragenic *HNF1B* pathogenic variants [58,59,65,68], challenging strict genotype–phenotype boundaries.

To date, ADTKD is classified into six genetically distinct subtypes based on the underlying molecular defect: ADTKD-*UMOD*, ADTKD-*MUC1*, ADTKD-*REN*, ADTKD-*HNF1B*, ADTKD-*SEC61A1*, and the exceptionally rare ADTKD-*DNAJB11* [65]. This latter subtype exhibits clinical commonalities with classic ADPKD and demonstrates significant clinical overlap with classic ADPKD through the presentation of bilateral renal and hepatic cysts; however, it represents a distinct phenotypic hybrid characterized by normal-sized or only mildly enlarged cystic kidneys and a slowly progressive course toward late-onset ESKD [66].

During diagnostic evaluation, large deletions encompassing the 17q12 region or the entire *HNF1B* gene are typically identified using chromosomal microarray (CMA) or through validated copy number variant (CNV) algorithms integrated into next-generation sequencing (NGS) data. Concurrently, high-depth NGS panels are applied to capture both *HNF1B* intragenic sequence variants and alterations in other alternative ADTKD loci. Within this modern diagnostic workflow, multiplex ligation-dependent probe amplification (MLPA) is no longer utilized as a primary screening tool, but remains a valuable secondary, orthogonal validation test to confirm ambiguous deletions. Crucially, because certain deep intronic or highly complex structural variants may still evade these standard testing architectures, a negative molecular result does not fully exclude a clinical diagnosis of ADTKD.

The main structural defects, extrarenal manifestations, and tailored high-depth sequencing strategies for typical and atypical ADPKD variations are integrated and contrasted in Table 1.

## 3. Pathophysiology

The molecular pathophysiology of ADPKD overlaps with related ciliopathies, as primary cilia act as polymodal sensors on the apical surface of renal epithelial cells [67,76]. When ciliary architecture or sensory cascades are altered, intracellular signaling pathways become profoundly dysregulated, initiating aberrant epithelial proliferation. An abnormal orientation of cell division, driven by disrupted planar cell polarity (PCP), leads to localized tubular dilatation, acting as the morphological precursor to cystogenesis [67,68,76,77].

PCP is not the sole signaling pathway involved. Cystogenesis is driven by a complex network of disrupted cascades, including altered intracellular calcium homeostasis, the acceleration of cyclic AMP (cAMP) signaling, constitutive activation of the mTOR pathway, and abnormal activation of the Wnt and MAPK/ERK pathways [67,76,77]. Together, these coordinated signaling defects disrupt cell differentiation, promote autonomous fluid secretion, and act as the primary drivers of progressive cyst growth.

**Figure 1:**
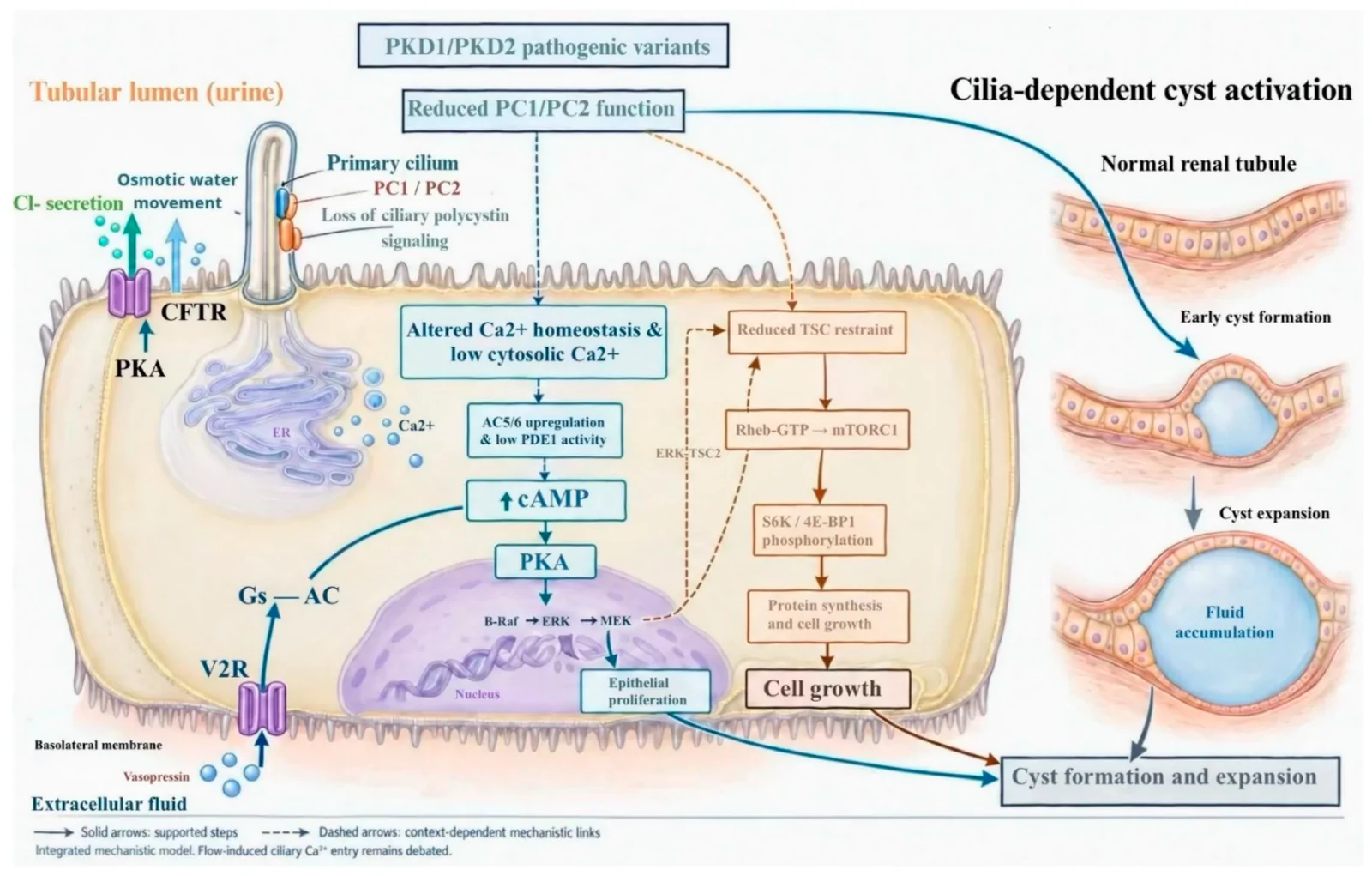
Molecular Signaling Cascades and Histological Evolution in ADPKD Cystogenesis. Pathogenic variants in *PKD1* or *PKD2* disrupt polycystin-1/2 (PC1/PC2) function at the primary cilium, leading to altered intracellular Ca^2+^ homeostasis and low cytosolic calcium levels. This loss of calcium restriction upregulates adenylyl cyclases (AC5/6) and reduces phosphodiesterase (PDE1) activity, triggering massive intracellular cyclic AMP (cAMP) accumulation. This cascade acts in synergy with basolateral vasopressin V2 receptor (V2R) activation. Elevated cAMP stimulates Protein Kinase A (PKA), driving apical Cl-secretion via CFTR channels and secondary osmotic water movement into the tubular lumen. Concurrently, PKA cross-talk activates the B-Raf/MEK/ERK pathway to drive epithelial proliferation, while mitogenic signaling inactivates the TSC1/TSC2 complex, leading to constitutive mTORC1 hyperactivation and accelerated cell growth. Morphologically (right), these coordinated disruptions drive the structural transformation of a normal renal tubule into expanding, fluid-filled isolated cysts. Solid arrows indicate supported steps; dashed arrows denote context-dependent mechanistic links. Abbreviations: AC, adenylyl cyclase; AC5/6, adenylyl cyclase isoforms 5 and 6; B-Raf, B-Raf proto-oncogene, serine/threonine kinase; Ca^2+^, calcium; cAMP, cyclic adenosine monophosphate; CFTR, cystic fibrosis transmembrane conductance regulator; Cl^−^, chloride; ER, endoplasmic reticulum; ERK, extracellular signal-regulated kinase; Gs, G protein alpha subunit; MAPK, mitogen-activated protein kinase; MEK, mitogen-activated protein kinase kinase; mTORC1, mechanistic target of rapamycin complex 1; PC1, polycystin-1; PC2, polycystin-2; PDE1, phosphodiesterase 1; PKA, protein kinase A; Rheb-GTP, Ras homolog enriched in brain guanosine triphosphate; TSC1, tuberous sclerosis 1 (hamartin); TSC2, tuberous sclerosis 2 (tuberin); V2R, vasopressin V2 receptor; 4E-BP1, eukaryotic translation initiation factor 4E-binding protein 1; S6K, ribosomal protein S6 kinase.

To provide a unified biochemical and spatial perspective of these complex networks, the interconnected intracellular cascades and the subsequent morphological transition from normal renal tubules to advanced fluid-filled cysts are structurally integrated in Figure 1.

### 3.1. Anatomical Origins and Histological Variations

Renal cysts arise from localized epithelial proliferation, causing initial tubular expansion, followed by the anatomical separation of the expanding cyst from the parent nephron and active, autonomous fluid secretion [74,76,78].

Cysts in patients with ADPKD are fluid-filled focal outgrowths from the renal epithelium that develop in only a minority of nephrons (approximately 1–5%), even though every somatic cell carries the same inherited germline pathogenetic variant. These cysts can emerge from all segments of the nephron, though they primarily develop within the distal tubules and collecting ducts. This focal and segmentally diverse pattern contrasts with autosomal recessive polycystic kidney disease (ARPKD), which predominantly affects the collecting ducts in a symmetric, bilateral pattern from fetal life, often precipitating neonatal respiratory compromise due to massive renal enlargement [78,79].

Rarely, glomerular cystic disease may represent the initial clinical presentation of ADPKD. However, these glomerular lesions are typically accompanied by scattered tubular cysts, a feature that helps distinguish early ADPKD from other inherited glomerulocystic diseases, such as those caused by HNF1B pathogenetic variants [17,79].

Histologically, cyst morphology can also differentiate ADPKD from contiguous gene syndromes. While cysts in the vast majority of classic ADPKD cases are lined by a single layer of bland, flattened epithelium, patients with the *TSC2/PKD1* CGS exhibit distinct renal cysts lined by multilayered, hyperplastic, and hobnail-like epithelial cells. These patients carry a high risk of developing renal angiomyolipomas due to the constitutive activation of the mTOR pathway [38,40,41]. Therefore, encountering such atypical histological features or rapid bilateral overgrowth reinforces the need for multigene panel sequencing to exclude complex locus rearrangements [54,55,56,57].

**Figure 2:**
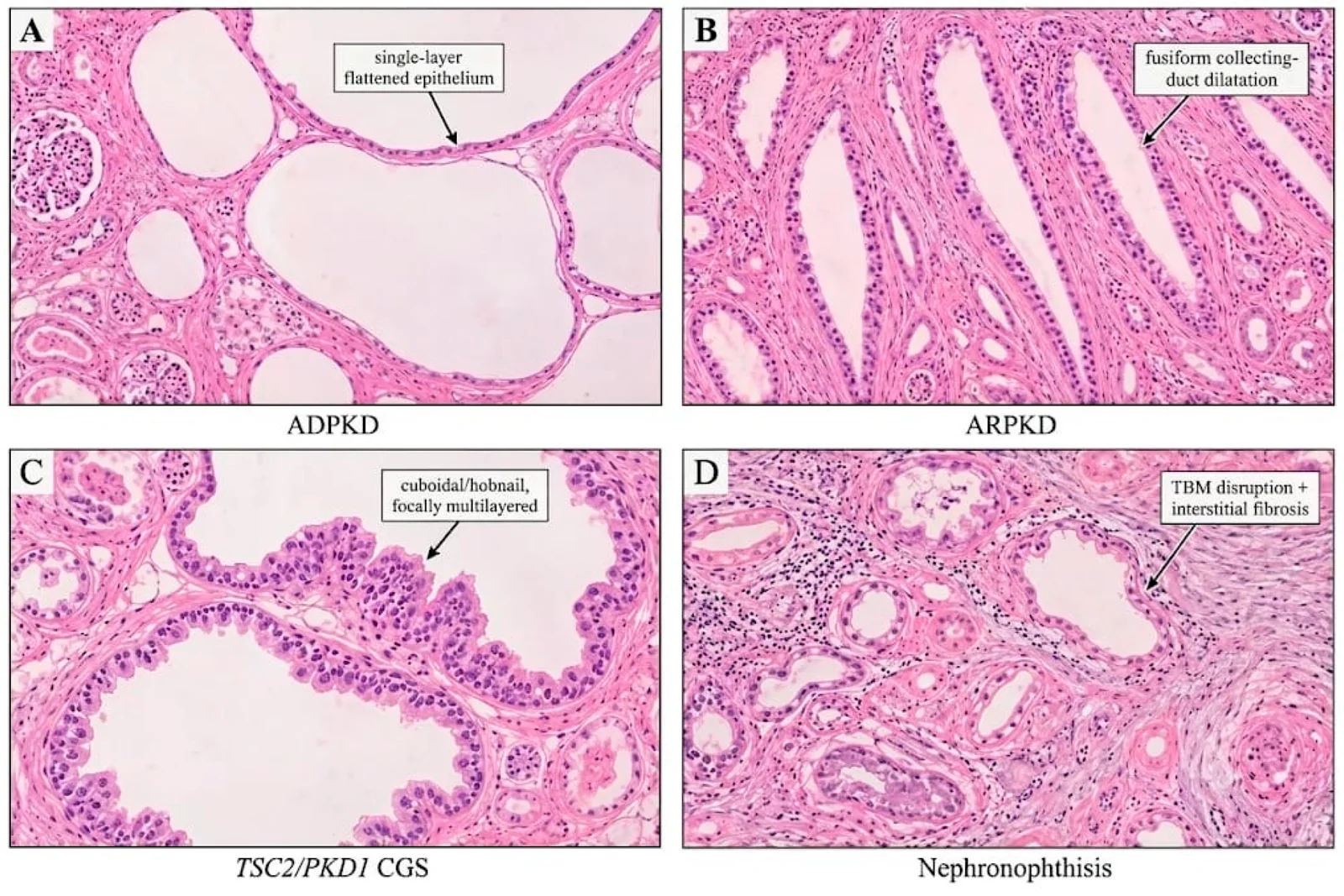
Comparative Renal Histology of ADPKD and Overlapping Genetic Nephropathies. Representative hematoxylin and eosin (H&E) stained sections showcasing distinct structural patterns: (**A**) ADPKD, presenting with large focal cysts lined by a characteristic single layer of flattened epithelial cells adjacent to preserved glomeruli; (**B**) ARPKD, illustrating classic, uniform fusiform dilatation of the collecting ducts radially arranged across the renal parenchyma; (**C**) *TSC2/PKD1* contiguous gene syndrome (CGS), characterized by atypical, severe cystic overgrowth lined by hyperplastic, cuboidal-to-hobnail, and focally multi-layered epithelial structures; and (**D**) Nephronophthisis, demonstrating marked tubular basement membrane (TBM) disruption, secondary tubular atrophy, and dense, progressive interstitial inflammatory fibrosis. Abbreviations: ADPKD, autosomal dominant polycystic kidney disease; ARPKD, autosomal recessive polycystic kidney disease; CGS, contiguous gene syndrome; TBM, tubular basement membrane; TSC2, tuberous sclerosis complex 2.

To structurally contrast these diagnostic variations, the distinct microscopic hallmarks separating classic ADPKD from alternative inherited cystic architectures and contiguous locus rearrangements are visually integrated in Figure 2.

### 3.2. Cellular Remodeling and the Warburg Effect

As ADPKD cysts expand, they completely detach from the tubular lumen and urinary space, functioning as independent, isolated cystic units [68,79].

This disruption occurs when the absolute level of functional polycystin-1 (PC1) or polycystin-2 (PC2) protein drops below a critical, tissue-specific threshold [68,79].

Consequently, a cascade of secondary alterations is triggered in cyst-lining epithelial cells, including a reversal of apical-basal polarity, compromised planar cell polarity (PCP), increased production of abnormal extracellular matrix components, and a rearrangement of cellular metabolism.

Similar to neoplastic tissues, the accelerated proliferation of cystic epithelial cells is sustained by a shift toward aerobic glycolysis, a metabolic reprogramming phenomenon known as the Warburg effect [77,80,81,82,83].

This metabolic shift provides the high-affinity energy and biosynthetic intermediates necessary to fuel continuous epithelial expansion and fluid secretion.

### 3.3. Molecular Triggers: The Two-Hit and Third-Hit Hypotheses

Although clinical ADPKD exhibits nearly 100% penetrance throughout life, cysts macroscopically affect only 3–5% of the total nephron population.

This striking focal distribution strongly supports the genetic two-hit hypothesis, which posits that the first hit is the inherited, germline heterozygous pathogenetic variant present across all renal cells, whereas the second hit is a subsequent, spontaneous somatic pathogenetic variant occurring in the remaining wild-type allele within an individual tubular cell. This somatic pathogenetic variant inactivates gene function, initiating clonal cyst formation. Thus, while ADPKD is clinically transmitted as an autosomal dominant genetic trait, it behaves as a recessive trait at the molecular and cellular level [82,83,84]. Emerging evidence suggests that specific genomic secondary-hit mechanisms involving DNA damage are implicated in focal cyst initiation.

Specifically, the formation of non-canonical Guanine-quadruplex (G4) structures can induce DNA strand breaks within the highly complex *PKD1* locus.

This G4-induced genomic instability promotes a high rate of local somatic mutagenesis, even in patients carrying mild or monoallelic germline variants. Recent next-generation sequencing (NGS) studies have confirmed unique somatic *PKD1* and *PKD2* pathogenetic variants isolated from individual cyst tissues, validating this mechanism. Disrupting G4 DNA stability has emerged as a promising strategy to mitigate local somatic mutagenesis and mitigate downstream disease acceleration [75,83,84,85].

The “third-hit” hypothesis describes the environmental, physiological, and ischemic triggers that accelerate cyst expansion after the molecular loss of the wild-type allele. A primary driver of this third hit is the hyperactivation of vasopressin (anti-diuretic hormone, ADH) signaling through V2 receptors in the collecting ducts, which increases intracellular cyclic AMP (cAMP) and stimulates both fluid secretion and cell proliferation. As these cysts grow, they compress the surrounding renal parenchyma, leading to localized tissue remodeling, microvascular damage, focal ischemia, and interstitial inflammation [86,87,88].

These coordinated genetic and microenvironmental alterations sequentially amplify tissue damage, promoting progressive interstitial fibrosis within non-cystic nephrons and culminating in a comprehensive decline in renal function [86,87,88].

## 4. Phenotypic Heterogeneity and Atypical ADPKD

The characterization of numerous genetic conditions presenting as “atypical ADPKD” has shifted the diagnostic paradigm of polycystic kidney disease.

This definition encompasses subsets of individuals displaying a macroscopically similar renal cystic phenotype but exhibiting a milder, distinct, or unusual clinical course [43,49]. Based on magnetic resonance imaging (MRI) assessments, approximately 10% of diagnosed ADPKD cases are classified as radiologically atypical (Mayo Clinic Class 2, subclassified into Class 2A for asymmetric or segmental patterns and Class 2B for bilateral sparse presentations). This subgroup frequently lacks conventional pathogenic *PKD1* or *PKD2* pathogenetic variants. Indeed, a recent cohort study of atypical ADPKD patients revealed that only 8.3% carried identifiable *PKD1* pathogenetic variants, whereas 41.7% harbored pathogenic variants in minor alternative loci such as *IFT140* and *ALG9*.

Although extrarenal involvement was documented in two-thirds of these atypical cases, progressive renal function decline was exceptionally rare.

These findings underscore the need to re-evaluate and refine how clinicians define and classify ADPKD spectrum disorders from both molecular and diagnostic imaging perspectives.

### 4.1. Minor Loci and Overlapping Digenic Phenotypes

*IFT140* variants produce large, sparse, and frequently exophytic renal cysts. Radiologically, these manifestations fit the Mayo Class 2 classification, exhibiting a bilateral but markedly asymmetric distribution, now formally designated under the updated KDIGO nomenclature as ADPKD-*IFT140*.

The renal prognosis for these patients is highly favorable; progression to kidney failure is rare, affecting fewer than 2% of individuals in seminal cohorts. Moreover, although specific systemic vascular anomalies have been described in association with IFT140 inactivation, clinical evidence remains limited, and current data do not support the use of vasopressin V2 receptor antagonists (e.g., tolvaptan) in this subpopulation.

Phenotypic complexity is frequently driven by digenic or synergistic modes of inheritance. Concomitant inheritance involving variants in *GANAB* combined with *COL4A3* or *COL4A5* can result in overlapping features between ADPKD and Alport syndrome, accelerating the rate of disease progression beyond the predictions of monoallelic models [72,73].

Similarly, exceptionally severe forms of ADPKD can arise from dual pathogenetic variants, where a classic *PKD1* pathogenetic variant co-occurs with variants in other cystic nephropathy loci, such as *HNF1B* or *PKHD1*.

### 4.2. Prenatal-Onset ADPKD and Early Infantile Presentations

Severe early-onset forms of ADPKD can manifest in utero or during early infancy, challenging the traditional view of ADPKD as an exclusively adult-onset condition. Prenatally, both ADPKD and autosomal recessive polycystic kidney disease (ARPKD) can present with markedly enlarged, hyperechogenic kidneys (HEK), occasionally accompanied by severe oligohydramnios and subsequent pulmonary hypoplasia. On prenatal ultrasound, increased corticomedullary differentiation (CMD) typically favors a diagnosis of early-onset ADPKD, whereas decreased or completely lost CMD is more characteristic of classic ARPKD [89,90,91,92]. These hyperechogenic renal alterations are commonly detected during routine anatomy scans at 18–20 weeks of gestation, though they can appear as early as 13–16 weeks [67,93,94,95,96]. Prenatal-onset ADPKD presentations displaying moderately enlarged hyperechoic kidneys with preserved or increased CMD can achieve favorable long-term pediatric outcomes, provided that amniotic fluid volume remains normal throughout gestation [95,96].

When standard chromosomal microarray testing yields non-diagnostic results in these infantile presentation cohorts, the rapid deployment of multigene targeted panels including *PKD1, PKD2, PKHD1*, and *HNF1B* is essential, as these four loci represent the most frequent genetic causes of isolated bilateral HEK. Nevertheless, whole-exome sequencing (WES) or whole-genome sequencing (WGS) continues to offer the highest diagnostic yield [69,97,98]. The critical impact of complex genetic interactions in early-onset disease is well-documented. In an infantile cohort where the onset of cystic kidney disease was recorded from the prenatal period up to 18 months of age, 17% of patients (5/30) carried two hypomorphic variants in the PKD1 gene, while 30% (9/30) carried a classic pathogenic variant on one allele alongside a likely hypomorphic variant on the other [69,70]. Interestingly, these genetic configurations represent de novo pathogenetic variants in up to 50% of cases [71].

While ADPKD is typically inherited as a monogenic dominant trait, severe very-early-onset (VEO) presentations are frequently driven by fully penetrant biallelic hypomorphic pathogenetic variants in *PKD1*, or by the combination of a classic *PKD1* truncation with an ultra-low penetrance modifier allele [69,72]. These complex biallelic configurations underscore the paramount importance of detailed family investigations to ensure accurate variant interpretation and comprehensive genetic counseling [67,95].

### 4.3. Syndromic Ciliopathies Mimicking ADPKD

The phenotypic spectrum of ADPKD overlaps with rare, syndromic renal ciliopathies. A prominent example is oral-facial-digital syndrome type 1 (OFD1), a rare X-linked dominant ciliopathy caused by pathogenetic variants in *OFD1* gene [74,75].

*OFD1* presents with a classic PKD-like renal phenotype characterized by bilateral macrocysts, co-occurring with systemic congenital malformations, including a cleft palate, cognitive impairment, and distinct digital anomalies (such as syndactyly, brachydactyly, or polydactyly) [74,75].

Because digital anomalies represent a highly consistent and accessible extrarenal manifestation across the broader spectrum of ciliopathies, their clinical identification suggests an underlying syndromic cystic kidney disease trait [74,75].

Beyond ciliary defects, non-ciliary syndromic disorders can also mimic early-onset polycystic phenotypes. Specifically, DICER1-related disease should be considered in the differential diagnosis of pediatric ADPKD presentations.

Although DICER1 syndrome is an established tumor-predisposition disorder [99], germline pathogenic variants can precipitate bilateral renal macrocysts or multilocular cystic nephroma during early childhood, structurally phenocopying ADPKD [100,101].

Clinically, DICER1-associated renal involvement can be differentiated by the absence of progressive, global kidney volume expansion and its frequent association with unique extrarenal neoplastic markers, such as pleuropulmonary blastoma, multinodular goiter, or thyroid malignancies, further highlighting the necessity of comprehensive genetic testing in atypical pediatric cohorts.

## 5. Clinical Manifestations, Complications, and Management

### 5.1. Renal Complications and Management

Early-stage ADPKD manifestations are insidiously progressive and often subclinical, typically presenting with impaired urinary concentrating capacity, nocturia, enuresis, microalbuminuria, proteinuria, and flank pain.

Gross hematuria is reported in approximately 10–14% of children before the age of 16 years, whereas polyuria and enuresis affect roughly half of all pediatric patients [102,103,104].

Pain can stem from mechanical cyst expansion, acute intralesional hemorrhage, or secondary urinary tract infections (UTIs) or nephrolithiasis.

Episodes of acute cyst hemorrhage are typically managed conservatively with bed rest, intensive hydration, and analgesia (e.g., paracetamol), usually resolving within 2–7 days; based on current pediatric consensus recommendations and the KDIGO 2025 guidelines, temporary discontinuation of renin-angiotensin-aldosterone system (RAAS) inhibitors is required during severe macroscopic hematuria to prevent acute kidney injury secondary to prerenal hemodynamic shifts or tubular obstruction by blood clots. Adjuvant agents like gabapentin may be added for refractory chronic pain [10,11,105,106,107]. Among patients with ADPKD and nephrolithiasis, uric acid stones are the most frequently encountered composition, followed by calcium oxalate crystals [10,11].

Among stone formers with ADPKD, uric acid stones are the most frequently encountered composition, followed by calcium oxalate stones [10,11].

UTIs in ADPKD follow standard pediatric protocols, with the caveat that asymptomatic bacteriuria should not be treated. For uncomplicated lower UTIs, first-line empiric options include nitrofurantoin, trimethoprim–sulfamethoxazole (TMP-SMX), or fosfomycin, guided by local resistance patterns, to ensure targeted pathogen eradication and avoid multidrug resistance, a mandatory urine culture must be obtained prior to initiating antibiotic therapy [105,106,107].

Clinically, a clear distinction must be made between these uncomplicated UTIs (cystitis) and a suspected cyst infection.

Uncomplicated UTIs typically present with isolated lower urinary tract symptoms, such as dysuria, frequency, and urgency, without systemic signs, and can be successfully eradicated with standard short-course hydrophilic antibiotics (approximately 7 days).

In contrast, a suspected cyst infection is a severe, tissue-invasive complication characterized by systemic symptoms including high fever, chills, localized flank or abdominal tenderness, and elevated inflammatory biomarkers (CRP and leukocytosis), often accompanied by negative urine cultures if the infected cyst does not communicate with the collecting system.

While short treatment courses (approximately 7 days) are sufficient for acute cystitis, a suspected cyst infection demands a prolonged course of 4–6 weeks utilizing highly lipid-soluble antibiotics capable of penetrating the cystic epithelium, such as TMP-SMX or fluoroquinolones, though the latter require caution in pediatrics due to potential musculoskeletal toxicity [105,106,107].

This extended duration is essential because standard hydrophilic antibiotics exhibit poor penetration into the cystic fluid; thus, clinical guidelines dictate 4–6 weeks of these lipid-soluble agents to actively cross the cystic epithelium and reach bactericidal thresholds within the cyst lumen.

In any child presenting with ADPKD and a concomitant UTI, a prompt renal ultrasound is indicated to exclude suppurative or obstructive complications, such as perinephric abscesses or infected cysts, that require immediate intervention [105,106,107].

Abdominal pain, nephrolithiasis, and proteinuria should otherwise be managed according to standard pediatric guidelines [10,11].

### 5.2. Extrarenal Manifestations and Systemic Involvement

The ubiquitous expression of polycystins across non-renal tissues characterizes ADPKD as a multisystem disorder although clinically evident extrarenal manifestations are exceedingly rare during childhood. In pediatric patients, screening for extrarenal phenotypes is strictly symptom-driven, and routine systemic screening is generally not indicated.

Individual genotypes heavily influence the onset, specific distribution, and severity of these extrarenal manifestations [8,10], dictating whether they present in youth or only later in adulthood.

The clinical relevance of the most common extrarenal findings in the pediatric population includes:

- Hepatic cysts: Exceptionally rare in pediatric cohorts. The early or extensive development of liver cysts in a child is highly unusual and should raise clinical suspicion for alternative fibrocystic liver diseases or autosomal recessive traits, rather than classic pediatric ADPKD.
- Pancreatic cysts: Virtually absent in children and selectively associated with classic *PKD1* pathogenetic variants in older patient cohorts.
- Intracranial aneurysms (ICA): The rupture of an ICA leading to subarachnoid hemorrhage (SAH) is the most severe potential complication, but it is extremely rare in pediatric patients. Proactive, non-invasive screening via magnetic resonance angiography is strictly reserved for high-risk pediatric cohorts with a documented family history of ADPKD accompanied by known early aneurysms or prior SAH events [108,109].
- Cardiac valvular disease: primarily manifesting as mitral valve prolapse, which can occasionally be detected in children but usually remains asymptomatic during youth.

Other systemic manifestations typically associated with adult ADPKD, such as splenic artery aneurysms, colonic diverticulosis, seminal vesicle cysts, and abnormalities in sperm parameters, are clinically irrelevant in the pediatric population and are generally restricted to adults or patients with complex overlapping dual pathologies [110,111].

Similarly, bronchiectasis and abdominal wall or inguinal hernias are rarely documented in classic pediatric cohorts. However, hyperlipidemia represents a metabolic comorbidity that may emerge early and should be monitored and treated according to standard cardiovascular guidelines to minimize long-term vascular risk [10,11].

Beyond classic ADPKD, alternative genetic loci associated with other cystic kidney diseases introduce distinct systemic phenotypes that must be clinically differentiated. Pathogenetic variants affecting *HNF1B* disrupt the classic clinical picture, leading to Autosomal Dominant Tubulointerstitial Kidney Disease (ADTKD-*HNF1B*) and its associated systemic manifestations.

Comprehensive 17q12 microdeletions disrupting *HNF1B* in the context of ADTKD are frequently tied to neurological abnormalities, including refractory epilepsy and autism spectrum disorders, whereas submicroscopic *HNF1B* point pathogenetic variants typically produce isolated kidney disease.

Importantly, clinicians should note that these neurodevelopmental phenotypes can also emerge in patients with isolated *HNF1B* pathogenic variants [68].

Requiring neuropsychiatric surveillance regardless of the specific deletion size.

Furthermore, structural *HNF1B* alterations cause Maturity-Onset Diabetes of the Young type 5 (MODY5) and are systemically characterized by early-onset diabetes mellitus, primary hyperparathyroidism, neonatal cholestasis, structural liver and pancreatic hypoplasia, and severe hyperuricemia.

### 5.3. Cardiovascular Phenotypes and Hemodynamic Monitoring in Pediatrics

Hypertension represents the most frequent cardiovascular complication in pediatric ADPKD, occurring in approximately 5–44% of cases.

It results from localized parenchymal ischemia induced by expanding cysts; this ischemic microenvironment triggers the premature activation of the systemic Renin-Angiotensin-Aldosterone System (RAAS) and drives pathologic sodium retention [112,113,114,115,116,117]. Adverse cardiovascular structural alterations, such as an increased left ventricular mass index (LVMI), can develop during childhood even when standard office blood pressure measurements remain within the normal range. A prospective, single-center longitudinal study focusing on pediatric patients, most of whom exhibited a preserved GFR identified through family screening, demonstrated that children presenting with a burden of more than 10 renal cysts experienced elevated rates of flank or back pain and early hypertension compared to those with a lower cyst count [118,119].

Multiple pediatric cohort studies have established a direct link between early hypertension and increased kidney volume calculated via ultrasound. Longitudinal monitoring of children and young adults has highlighted glomerular hyperfiltration and rapid kidney enlargement as primary predictors of long-term disease progression [50,104,120]. Similarly, an impaired urinary concentrating ability represents a well-established functional hallmark of early ADPKD manifest in both pediatric and adult cohorts.

The optimal method to normalize TKV growth in growing children (utilizing either height or body surface area adjustments) remains a subject of ongoing debate, and no validated risk prediction software tools currently exist to identify which asymptomatic children will progress rapidly. Furthermore, there are no approved disease-modifying drugs specifically labeled for pediatric ADPKD.

To mitigate long-term cardiovascular risks, current consensus guidelines suggest an annual office blood pressure measurement starting strictly at age 5 years in all children and adolescents diagnosed with or at risk for ADPKD.

In pediatric patients presenting with very-early-onset (VEO) or early-onset (EO) disease, or whenever an office BP measurement tracks above the 75th percentile for age, sex, and height, a 24-h ambulatory blood pressure monitoring (ABPM) should be performed annually (applicable for children ≥ 5 years of age and ≥120 cm in height). Regular echocardiography should also be deployed to screen for the development of left ventricular hypertrophy [121,122].

The strict therapeutic blood pressure targets in pediatric ADPKD are established at ≤50th percentile for age, sex, and height, or ≤110/70 mmHg in older adolescents. To achieve these targets, Angiotensin-Converting Enzyme (ACE) inhibitors or Angiotensin II Receptor Blockers (ARBs) are the first-line pharmacotherapy, offering superior nephroprotection particularly in the presence of concomitant proteinuria [113,122].

## 6. Diagnostic Criteria, Biomarkers, and Risk Stratification

### 6.1. Pediatric Diagnosis, Ultrasonographic Challenges and Advanced Imaging Limitations

A global consensus remains unestablished regarding systematic screening protocols for asymptomatic, at-risk children.

This clinical caution is primarily rooted in the psychosocial impact, emotional distress, and potential insurance discrimination associated with an early genetic diagnosis. Consequently, there is a strong ethical consensus toward delaying predictive testing in asymptomatic minors until they can provide informed consent as adults. When testing is requested by parents or mature minors after thorough genetic counseling, the primary clinical objective is not to initiate adult-centric disease-modifying therapies. Instead, early detection targets the screening and treatment of early complications, particularly pediatric hypertension and microalbuminuria, which are critical to slowing long-term renal decline.

To establish a clinical diagnosis in this population, international pediatric consensus recommendations [11] and the updated 2025 KDIGO Clinical Practice Guideline [10] dictate specific age- and family-dependent criteria that overcome the suboptimal sensitivity of adult-validated metrics, such as the Pei–Ravine criteria, in individuals under 15 years of age. In a child with a known family history of ADPKD, the presence of even a single solitary renal cyst (>1 cyst) detected via high-resolution renal ultrasonography (US) is highly suggestive and considered sufficient for a clinical diagnosis.

Because isolated simple benign cysts are exceptionally rare in the pediatric population, defined as round, thin-walled, anechoic lesions lacking internal septa or Doppler flow within an otherwise normal parenchyma, even a single solitary cyst in an at-risk child necessitates longitudinal monitoring of cyst count, size, and morphological characteristics [123,124,125].

**Figure 3:**
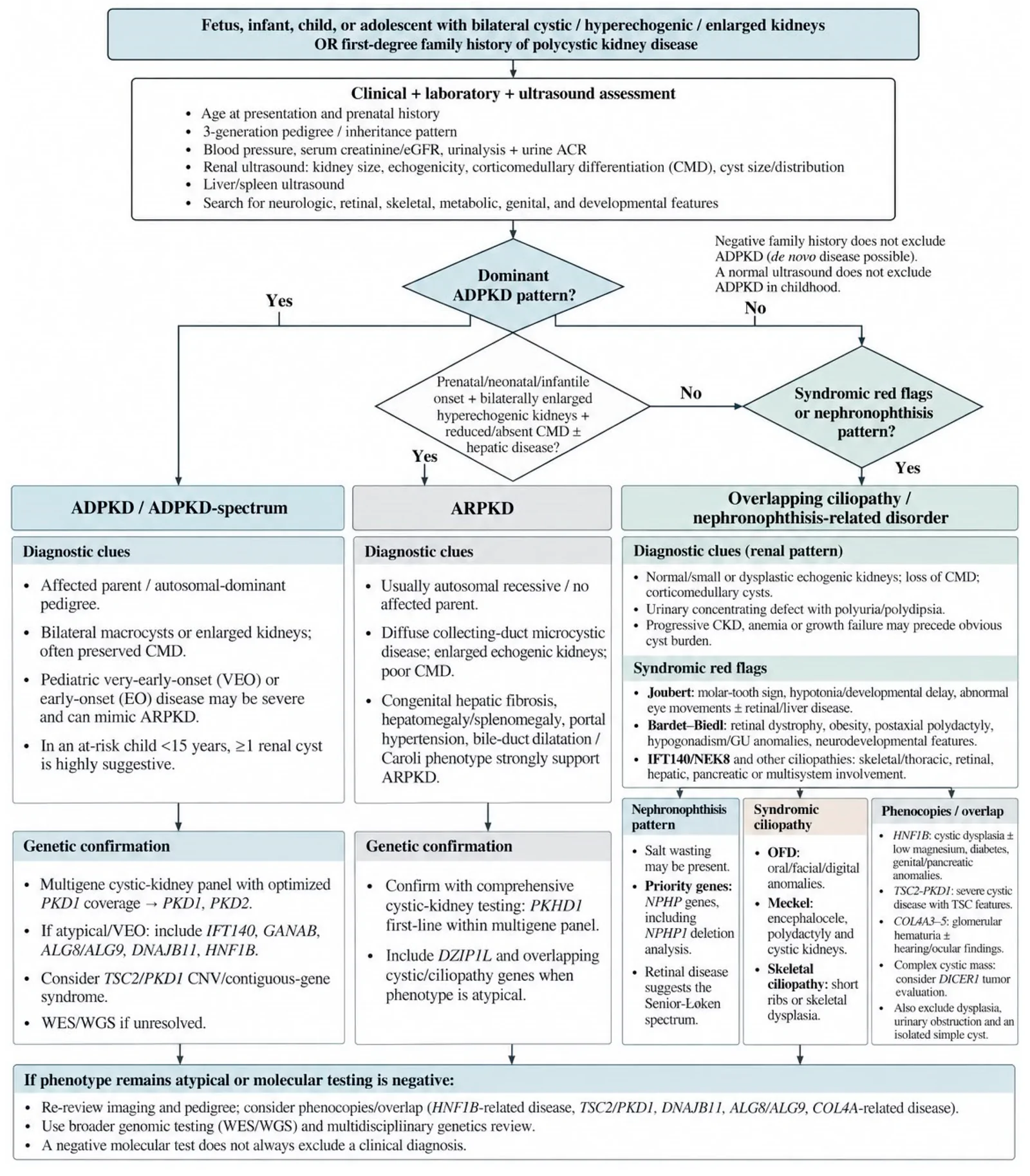
Pediatric Cystic Kidney Disease Diagnostic Framework. Clinical flowchart for the evaluation of fetuses, infants, and children presenting with bilateral cystic or hyperechogenic kidneys, or a positive family history. Based on pedigree analysis, parental ultrasound screening, and key imaging features, such as preserved versus lost corticomedullary differentiation (CMD), patients are stratified into three main diagnostic pathways: ADPKD/ADPKD-spectrum, classic ARPKD, or overlapping ciliopathies/nephronophthisis-related disorders. The bottom panel provides secondary evaluation workflows for atypical or molecularly unresolved cases. Abbreviations: ACR, albumin-to-creatinine ratio; ADPKD, autosomal dominant polycystic kidney disease; ARPKD, autosomal recessive polycystic kidney disease; BP, blood pressure; CKD, chronic kidney disease; CMD, corticomedullary differentiation; CNV, copy number variation; eGFR, estimated glomerular filtration rate; GU, genitourinary; HNF1B, hepatocyte nuclear factor 1-beta; MRI, magnetic resonance imaging; NGS, next-generation sequencing; OFD, oral-facial-digital syndrome; TSC, tuberous sclerosis complex; VEO/EO, very-early/early-onset; WES, whole-exome sequencing; WGS, whole-genome sequencing.

To systematically guide clinicians through the initial clinical evaluation, imaging-based differential diagnosis, and targeted genetic testing strategies of these overlapping pediatric phenotypes, a comprehensive clinical management workflow is illustrated in Figure 3.

The diagnostic approach is more complex in the absence of a known family history, where incidental finding of a solitary cyst or bilateral hyperechogenic kidneys (HEK) requires systematic familial evaluation. In line with the 2025 KDIGO guidelines, renal US should be performed on the parents; if the parents are under 40 years of age and display negative imaging, screening must be extended to the grandparents to rule out an inherited trait before concluding a de novo pathogenetic variant, which accounts for approximately 10–25% of cases [124,125,126].

Furthermore, both the 2025 KDIGO guidelines and pediatric consensus statements strongly recommend confirmatory genetic testing in children when imaging findings remain equivocal or atypical, when family history is absent, or when a definitive differentiation from overlapping syndromic ciliopathies is mandatory.

While modern high-resolution US can reveal minute early cysts (2–3 mm) or asymmetric lesions [50,94], no validated standalone imaging criteria currently exist to confirm or exclude ADPKD in pediatric patients lacking a positive family history, particularly when a milder underlying genotype is present [123,127,128].

This limitation highlights the importance of screening the parents of an affected child; however, if the parents are relatively young, their negative imaging should be interpreted cautiously due to age-dependent penetrance [10,11], which can leave a subset of genetically positive parents appearing temporarily unaffected.

Once a diagnosis is confirmed or highly suspected, longitudinal surveillance must be initiated, focusing heavily on early cardiovascular risk stratification. To optimize long-term outcomes, the 2025 KDIGO guideline introduces rigorous blood pressure monitoring protocols, strongly emphasizing the routine deployment of 24-h ambulatory blood pressure monitoring (ABPM) beginning at 5 years of age. This assessment should be performed annually in children presenting with established hypertension or early-onset/very early-onset (EO/VEO-ADPKD) phenotypes, and every 2–3 years in other confirmed or at-risk children, utilizing first-line renin-angiotensin system inhibitors (RASi) for blood pressure control. Beyond blood pressure monitoring, advanced imaging modalities face significant implementation hurdles in pediatric care.

While MRI provides gold-standard volumetric precision that correlates tightly with ultrasound-derived TKV in children, its widespread utility in infants remains heavily constrained by motion artifacts and the frequent necessity for sedation.

Similarly, Computed Tomography (CT) imaging offers reliable volumetric data, but its regular deployment is avoided in pediatric cohorts to minimize cumulative ionizing radiation risk.

Consequently, adult imaging-based prognostic frameworks, such as the Mayo Imaging Classification (MIC), which integrates height-adjusted TKV (htTKV) kinetics to identify rapid progressors are restricted to patients aged 15 years or older and cannot be utilized for risk stratification in younger children.

Furthermore, these frameworks fail to capture risk in patients carrying pathogenic variants in minor atypical genes (such as *IFT140* or *GANAB*), or in *DNAJB11*-associated disease. These atypical conditions, which can occasionally manifest structural changes in childhood, are characterized by relatively few, small cysts and near-normal TKV despite progressive interstitial fibrosis [50,129,130,131], further underscoring the need for pediatric-specific diagnostic and prognostic approaches.

### 6.2. Prognostic Predictive Models Across the Lifespan

The clinical presentation of ADPKD is highly heterogeneous, ranging from severe, early-onset variants to insidious adult forms, which necessitates distinct age-specific risk models. Approximately half of affected individuals will reach kidney failure by approximately 60 years of age, exhibiting an accelerated estimated glomerular filtration rate (eGFR) decline averaging 4–6 mL/min/1.73 m^2^ per year [1,2,3,4]. For Very-Early-Onset (VEO-ADPKD), a definitive prenatal diagnosis requires the ultrasound documentation of bilateral, markedly enlarged, hyperechoic kidneys exceeding 2 standard deviations (SD) above the gestational mean, frequently complicated by concomitant oligohydramnios.

In infants up to 18 months of age, diagnostic criteria include enlarged, palpable kidneys accompanied by at least one systemic sign, such as blood pressure above the 95th percentile, an eGFR < 90 mL/min/1.73 m^2^, or persistent proteinuria [132,133]. For Early-Onset ADPKD (EO-ADPKD) presenting between 18 months and 15 years, the presence of large or palpable kidneys combined with hypertension, reduced eGFR, or persistent proteinuria indicates an aggressive phenotype [132,133]. A rapid, atypical decline in GFR before adulthood represents an important warning sign, prompting the consideration of complex genetic backgrounds, such as biallelic or compound heterozygous *PKD1* pathogenetic variants, hypomorphic PKD1 variants, or additional modifier variants in alternative ciliary genes [27,97,98]. Typically, individuals harboring classic *PKD1* pathogenetic variants reach stage 5 CKD around 60 years of age, whereas those with *PKD2* variants progress on average two decades later. Following diagnosis, prognostic risk assessment guides clinical management. The Predicting Renal Outcome in Polycystic Kidney Disease (PROPKD) score integrates both clinical and genetic data to categorize patients according to their future risk of ESKD, although its predictive value is primarily validated in individuals aged 35 years or older. In contrast, for pediatric cohorts, the Leuven Imaging Classification (LIC) is the first system specifically developed for early risk stratification [115,134].

Early-life risk factors, including pediatric hypertension, proteinuria, gross hematuria, and a burden of more than ten cysts per kidney, closely correlate with accelerated TKV growth and poorer renal outcomes later in life [115,135,136,137].

Recent longitudinal data suggest that the onset of hypertension in the mid-twenties to early thirties, rather than after age 35 as originally weighted in the PROPKD score, more accurately predicts early kidney failure, potentially justifying a future revision of this prognostic threshold [116,138].

In adult cohorts, comprehensive risk profiles associate a poorer long-term prognosis with male sex, higher Body Mass Index (BMI), macroscopic hematuria episodes before age 30, high dietary sodium intake, reduced baseline renal perfusion, and an increased height-adjusted TKV (htTKV), as established by the Consortium for Radiologic Imaging Studies of Polycystic Kidney Disease (CRISP) study [135,139]. Beyond these clinical and imaging predictors, novel biofluid-based biomarkers, such as urinary monocyte chemoattractant protein-1 (MCP-1) and neutrophil gelatinase-associated lipocalin (NGAL), are increasingly evaluated to monitor early interstitial inflammation and predict rapid disease progression before significant eGFR decline occurs.

The main clinical, imaging, and molecular criteria for ADPKD diagnosis and risk stratification are summarized and contrasted in Table 2.

### 6.3. Current Evidence, Controversies, and Knowledge Gaps

The clinical management of PKD and related ciliopathies in children currently faces a significant gap: while genetic sequencing allows for precise molecular diagnoses, specific prognostic tools and targeted therapies validated for pediatric populations remain unavailable.

This lack of child-specific frameworks frequently forces clinicians to extrapolate guidelines and therapeutic targets from adult trials, overlooking the unique hemodynamic, metabolic, and somatic growth dynamics that characterize childhood development. The disconnect between molecular advances and clinical practice involves complex methodological and ethical challenges. These include ongoing debates on how to normalize kidney volume during active body growth, the psychosocial impact of predictive genetic screening in asymptomatic minors, and distinct toxicological constraints when managing disease complications in young patients. To provide a clear synthesis of these current barriers and address the fragmentation of data in the literature, the core controversies and knowledge gaps are consolidated and contrasted in Table 3.

## 7. Therapeutic Advances and Future Directions

Tolvaptan remains the only drug approved by the Food and Drug Administration (FDA) and the European Medicines Agency (EMA) to slow cyst growth and delay the decline of kidney function in adults with rapidly progressive ADPKD [142,143]. Tolvaptan acts as a selective, high-affinity vasopressin V2 receptor antagonist. By blocking this receptor, the drug lowers intracellular cyclic AMP (cAMP) levels, thereby reducing cAMP-dependent protein kinase activation, epithelial cell proliferation, and chloride-driven fluid accumulation within the cysts. The seminal TEMPO 3:4 trial showed that this targeted approach can slow the annual increase in total kidney volume (TKV) and attenuate the loss of estimated glomerular filtration rate (eGFR) in adult patients at high risk of progression. However, its deployment in pediatric cohorts remains highly cautious. Tolvaptan is not yet approved for pediatric ADPKD due to a lack of long-term safety data and ongoing concerns regarding its impact on growth and development. Although early results from a recent phase 3 pediatric study suggest that V2 receptor antagonism might help curb progressive kidney enlargement and preserve renal function in young patients, these findings necessitate validation in larger, long-term multi-center trials before routine pediatric use can be safely considered [144,145,146].

Because vasopressin signaling drives cystogenesis, the use of vasopressin analogues (such as desmopressin) for nocturnal enuresis is contraindicated in children diagnosed with, or suspected of having, ADPKD [10].

### 7.1. Statin Therapy and Metabolic Interventions

For atypical ADPKD phenotypes (e.g., *IFT140* or *ALG9* variants), reliable biomarkers to forecast disease progression remain unestablished due to the scarcity of large-scale longitudinal data. Management for these atypical cohorts focuses primarily on symptomatic relief and standard nephroprotection rather than targeted molecular protocols [10,154].

Among alternative medications explored in younger populations, pravastatin has emerged as a therapeutic candidate. In pediatric clinical trials, pravastatin attenuated the rate of increase in height-adjusted TKV (htTKV) [147], although it did not modify baseline cardiac parameters, microalbumin excretion, or global renal function [147,148]. This suggests a potential structural benefit on vascular and cystic remodeling that requires further longitudinal investigation [147,148].

However, because these structural findings did not translate into a clear improvement or preservation of renal function, pravastatin is currently not considered an established disease-modifying therapy for pediatric ADPKD.

Beyond pharmacological interventions, a potential connection between glucose metabolism and ADPKD progression is being explored.

Experimental data from preclinical in vitro and animal models suggest that cystic epithelial proliferation is sustained by accelerated aerobic glycolysis (the Warburg effect), showing hypersensitivity to glucose availability.

Preliminary pediatric clinical observations indicate that children with ADPKD can exhibit a high index of insulin resistance (elevated HOMA-IR) even when maintaining a normal Body Mass Index (BMI) [149]. While this suggests a potential intrinsic metabolic vulnerability associated with polycystin dysfunction, a direct causal link to accelerated cystogenesis or accelerated GFR decline in childhood remains unestablished. Consequently, early clinical attention to pediatric obesity, metabolic comorbidities, and insulin sensitivity is highly recommended as a standard nephroprotective and cardiovascular risk-reduction strategy, rather than a validated disease-modifying protocol to curb cyst kinetics in youth.

### 7.2. Emerging Molecular Targets

Ongoing clinical trials are assessing novel small molecules targeting alternate pathways, including the mechanistic target of rapamycin (mTOR) signaling, tyrosine kinases, and altered cellular metabolism.

At the cellular level, polycystin-1 and the TSC1/TSC2 complex jointly repress the mTOR pathway to maintain tight control over cell proliferation.

When this regulatory mechanism is genetically impaired, constitutive activation of mTOR promotes rapid cyst formation and growth, rendering this pathway a critical therapeutic target. These investigations increasingly rely on TKV as a primary surrogate endpoint, aligning with the KDIGO 2025 Clinical Practice Guidelines, which formalize TKV as a validated biomarker for risk stratification and treatment decision-making [56,140,150].

Real-world data support the earlier initiation of targeted therapy in patients categorized at high genetic and imaging-based risk, establishing genotype-driven risk stratification as a fundamental element of ADPKD management.

Multi-targeted drugs, such as Tepotinib (a selective MET receptor tyrosine kinase inhibitor) and Pasireotide (a somatostatin analogue), are under evaluation as potential therapeutic options in ADPKD and related syndromes, including the *PKD1/TSC2* contiguous gene syndrome (CGS). However, due to undetermined safety profiles, these agents are not recommended for pediatric patients [56,140,150,151]. Pediatric trials frequently emphasize that early-onset hypertension and persistent proteinuria represent the key warning signs for rapid disease progression, strengthening the clinical rationale for early molecular detection and intensive blood pressure control in youth [141,155].

### 7.3. Advanced Biological Approaches

Gene-editing platforms (e.g., CRISPR/Cas9 systems) and RNA-based therapies (such as antisense oligonucleotides or microRNA modulators) are currently under evaluation for monogenic conditions like ADPKD. These approaches seek to either repair the primary genetic defect or re-regulate the downstream expression of polycystins and modifying ciliary genes. However, translating these therapeutics into clinical practice remains constrained by challenges in targeted organ delivery and off-target genomic effects [152,153]. Progress in vehicle design and high-fidelity editing mechanisms is necessary to transition these approaches from preclinical models to clinical applications [152,153]. The primary molecular mechanisms, ongoing clinical trials, and therapeutic recommendations for typical and atypical ADPKD are summarized in Table 4.

## 8. Conclusions and Future Perspectives

ADPKD exhibits complex genetic and phenotypic heterogeneity that challenges historical diagnostic and therapeutic paradigms. A precision medicine framework incorporating high-throughput genomic testing (such as customized next-generation sequencing panels and whole-genome sequencing), refined imaging classifications, and personalized risk-based interventions is required. The integration of prenatal and pediatric longitudinal data, a deeper understanding of somatic second-hit mechanisms (including G-quadruplex-induced genomic instability), and the inclusion of novel disease-associated loci (such as *IFT140*, *NEK8*, and *COL4A5*) into routine diagnostic workflows are reshaping the management of the ADPKD-spectrum phenotype.

Several multi-parametric prognostic tools are undergoing clinical refinement, including the Predicting Renal Outcome in Polycystic Kidney Disease (PROPKD) score, the Mayo Imaging Classification (MIC), and the global consensus outcomes defined by the Standardised Outcomes in Nephrology-Polycystic Kidney Disease (SONG-PKD) initiative [56,136,140,150,156].

These frameworks aim to integrate molecular, volumetric, and clinical indicators to identify patients at high risk for rapid progression, facilitating targeted therapeutic strategies before irreversible parenchymal damage ensues.

Promising biofluid biomarkers, specifically plasma copeptin and urinary MCP-1, offer robust predictive utility in adult populations, underscoring the necessity for extensive clinical trial validation in pediatric and very-early-onset cohorts.

Furthermore, next-generation imaging methodologies are emerging to refine risk stratification. Automated quantitative cyst segmentation and magnetic resonance texture analysis, powered by machine learning algorithms, enable the detection of minute, sub-radiological structural alterations across both expanding cysts and adjacent non-cystic renal parenchyma. Because the extensive phenotypic variability of the disease precludes a single universally applicable prognostic marker, future clinical practice must deploy a multi-modal combination of high-depth genotyping, TKV kinetics, validated risk scores, and biofluid biomarkers to reliably segregate fast progressors requiring early, intensive intervention from patients with a benign clinical course.

## Figures and Tables

**Table 1:** Genotype–Phenotype Correlations and Differential Diagnosis of ADPKD and Overlapping Ciliopathies.

Gene/Group	Type of Genetic Alteration	Inheritance Transmission	Typical Age at Presentation	Pediatric vs. Adult Phenotypes	Typical Renal Phenotype	Extrarenal Manifestations	Major Imaging Features	**Most Appropriate** **Molecular Diagnostic Approach**	**References**
*PKD1*	Loss-of-function (truncating, missense), hypomorphic variants, or deep intronic pathogenic variants.	AD; ~85% of classic cases	Typically 3rd–4th decade; VEO forms present prenatally/at birth.	Pediatric: 2–5% of cases; ~3% aggressive/VEO with altered utero kidney development. Adult: Progressive TKV expansion, ESKD around 55–60 years. Truncating and 5′ variants drive earlier ESKD than 3′ modifications.	Numerous bilateral cysts, rapid TKV expansion, progressive GFR decline; ESKD manifests around 55–60 years. Truncating variants link to a more severe course, whereas 5′ variants associate with earlier ESKD than 3′ modifications.	Biallelic hypomorphic configurations drive very-early-onset (VEO) or ARPKD-like phenotypes. Ubiquitous extrarenal polycystin expression causes multi-organ systemic involvement including liver cysts, pancreatic cysts, and blood vessel aneurysms (such as intracranial aneurysms), which are highly penetrant in adulthood but exceptionally rare in children.	Numerous bilateral, progressive fluid-filled renal cysts; massive, symmetric total kidney volume (TKV) expansion.	Locus-specific Long-Range PCR (LR-PCR) followed by high-depth NGS or WGS to circumvent 6 highly homologous pseudogenes (exons 1–33).	[12,14,15,17,27,28,29,30,31,32,33,69,70,71]
*PKD2*	Loss-of-function variants (half are unique/family-private).	AD; ~15% of classic cases	4th–5th decade; exceptionally rare in youth.	Pediatric: Asymptomatic; cysts are absent or single. Adult: Milder clinical course; delayed cystogenesis and fewer cysts; ESKD manifests ~2 decades later than PKD1.	Cysts are generally fewer with a later onset; progression to ESKD occurs approximately two decades later than in PKD1 cohorts on average.	Encodes polycystin-2 (a calcium-permeable TRP channel); exhibits a milder but clinically overlapping systemic phenotype with PKD1, including liver cysts, pancreatic cysts, and vascular aneurysms that manifest later in life	Bilateral renal cysts with slower kinetics and milder, delayed TKV expansion compared to PKD1 cohorts.	Standard multigene Next-Generation Sequencing (NGS) panels or Whole-Exome Sequencing (WES).	[12,14,17,32,33,34,35]
*GANAB*	Monoallelic loss-of-function variants.	AD; atypical spectrum	Adulthood; atypical or incidental presentation.	Pediatric: Asymptomatic; no pediatric clinical phenocopy reported. Adult: Mild-to-moderate, slowly progressive renal cystic disease; preserved global kidney function.	Fewer renal cysts, mild-to-moderate kidney enlargement, and slowly progressive course.	Prominent, penetrant liver cysts (ADPLD phenotype); crucial role in polycystin-1 maturation and traffic.	Fewer, small, or radiologically atypical bilateral renal cysts; stable or minimally increased total kidney volume	Comprehensive multigene NGS panel including alternative polycystic liver and ER-associated loci.	[10,12,72]
*DNAJB11*	Monoallelic mutations in an ER-localized co-chaperone of GRP78.	AD; ADTKD spectrum	Strictly adult-onset; late-onset ESKD.	Pediatric: Absent; no childhood presentation documented. Adult: Unique hybrid ADPKD/ADTKD phenotype; severe interstitial fibrosis despite mild cyst burden.	Few small cysts, normal or near-normal TKV, marked interstitial fibrosis, and late-onset ESKD.	Represents a phenotypic hybrid between ADPKD and ADTKD; encodes an ER-localized co-chaperone of GRP78.	Few small bilateral cysts, marked tubulointerstitial fibrosis, normal or near-normal total kidney volume (preserved TKV).	Integrated multi-gene NGS panel covering both structural cystic disease and tubulointerstitial kidney disease loci.	[50,66]
*ALG8*	Monoallelic loss-of-function variants in ER-associated glycosylation loci.	AD; atypical spectrum	Adulthood (AD); Neonatal/Infantile period (AR).	Pediatric: Asymptomatic in AD; severe systemic metabolic traits in AR. Adult: Mild, late-onset radiologically atypical ADPKD or ADPLD phenocopy.	Few, small bilateral renal cysts, mild kidney enlargement, and non-progressive course	Prominent and penetrant liver cysts (ADPLD-like phenotype); biallelic variants cause severe congenital disorder of glycosylation (*ALG8*-CDG).	Multiple renal and hepatic cysts typically characterized by a mild course, sparse distribution, and preserved kidney function.	Comprehensive multigene NGS panel or Whole-Exome Sequencing (WES).	[10,11,58]
*ALG9*	Monoallelic loss-of-function variants in ER-associated glycosylation loci.	AD; atypical spectrum	Adulthood (AD); Neonatal/Infantile period (AR).	Pediatric: Asymptomatic in AD; severe systemic development/neurological traits in AR. Adult: Mild, late-onset radiologically atypical ADPKD phenocopy.	Renal and hepatic cysts; typically presents as a mild, late-onset, or radiologically atypical phenocopy.	Encodes an ER glycoprotein involved in protein N-glycosylation and ciliary trafficking of polycystins.	Few small, sparse, or radiologically atypical bilateral renal and hepatic cysts with stable TKV.	Comprehensive multigene NGS panel or Whole-Exome Sequencing (WES).	[10,11,58]
*IFT140*	Monoallelic (AD) vs. Biallelic (AR) loss-of-function variants.	Monoallelic: AD; Biallelic: AR	Strictly adult-onset (AD); Prenatal/Neonatal (AR).	Pediatric: Asymptomatic in AD; severe syndromic features in AR. Adult: Atypical mild ADPKD-*IFT140* phenotype; favorable prognosis with <2% progressing to kidney failure.	Monoallelic: Large, sparse, frequently exophytic cysts with moderate TKV and relatively preserved kidney function. Third most frequent cause of ADPKD-spectrum disease.	Biallelic: Causes severe syndromic recessive ciliopathies (e.g., Jeune asphyxiating thoracic dystrophy, Mainzer–Saldino syndrome, cranioectodermal dysplasia).	AD: Large, sparse, frequently exophytic renal cysts with relatively preserved kidney function. AR: Small, diffusely microcystic/dysplastic kidneys.	Targeted multigene NGS panels or Whole-Genome Sequencing (WGS).	[42,43,44,45,46,47]
*NEK8*	Monoallelic pathogenic variants (AD) vs. Biallelic variants (AR).	Monoallelic: AD; Biallelic: AR	Early childhood (AD); Perinatal lethal (AR).	Pediatric: Rapidly progressive VEO-ADPKD (AD); lethal systemic dysplasia (AR). Adult: Advanced chronic kidney disease tracking from childhood.	Monoallelic: Moderately enlarged kidneys with rapid disease progression toward VEO-ADPKD.	Biallelic: Strictly linked to severe, lethal multisystem syndromic ciliopathies (renal-hepatic-pancreatic dysplasia).	Moderately to severely enlarged kidneys with rapid, aggressive cyst kinetics and progressive parenchymal architectural distortion.	Multigene NGS panel covering VEO-ADPKD and severe syndromic ciliopathies.	[54,55,56,57]
*HNF1B*	Structural 17q12 microdeletions or submicroscopic point pathogenic variants.	AD; ADTKD spectrum	Prenatal period through young adulthood; highly variable penetrance.	Pediatric: Prenatal bilateral hyperechoic kidneys (HEK), renal dysplasia, and early-onset neurodevelopmental traits. Adult: Progressive interstitial fibrosis, diabetes.	Renal cysts, dysplasia, and structural abnormalities; represents a distinct phenotypic hybrid between ADPKD and ADTKD.	Driven by full gene deletions (with neighboring genes in 17q12) or intragenic missense/truncating loss-of-function variants. Neurodevelopmental phenotypes (autism, learning difficulties) can occur in both groups, though more prevalent in 17q12 microdeletions often associated with MODY5 diabetes. Crucially, both deletions and intragenic variants can cause the entire phenotypic spectrum.	Cortical renal cysts, unilateral or bilateral renal hypoplasia/dysplasia, normal or small-sized kidneys with marked tubulointerstitial fibrosis.	Chromosomal Microarray (CMA) or NGS-based CNV algorithms to detect large 17q12 or *HNF1B* deletions; high-depth NGS panels/WGS for intragenic variants. MLPA is reserved as a secondary orthogonal validation tool for ambiguous deletions	[58,59,65,68]
*PKHD1*	Loss-of-function variants in fibrocystin.	Biallelic: AR; Monoallelic: AD-like	Prenatal or neonatal period (Classic ARPKD).	Pediatric: Severe neonatal respiratory compromise, massive bilateral kidney enlargement tracking in infancy. Adult: Caroli disease or isolated portal HTN.	Biallelic: Classic ARPKD presentation with massive kidney enlargement and symmetric collecting duct ectasia. Implicated in complex VEO/EO “transition” phenotypes between ARPKD and ADPKD.	Biallelic pathogenic variants characteristically cause congenital hepatic fibrosis (CHF) driven by ductal plate malformations, presenting with portal hypertension and splenomegaly later in childhood.	Massive, symmetric bilateral kidney enlargement with diffuse collecting duct ectasia; loss of corticomedullary differentiation (CMD).	Comprehensive multigene NGS panels or targeted duplication/deletion testing.	[58,59,69]
*COL4A3–A5*	Missense, splicing, or truncating variants in type IV collagen.	AD, AR, or X-linked	Childhood to young adulthood.	Pediatric: Persistent microhematuria, non-nephrotic proteinuria, or incidental renal cysts. Adult: Progressive CKD, Alport syndrome phenotypic traits.	Bilateral renal cysts accompanied by microhematuria or proteinuria; can mimic or overlap with Alport syndrome.	Characteristically associated with progressive sensorineural hearing loss and specific ocular diseases, including anterior lenticonus and maculopathy. Digenic or synergistic inheritance patterns reported (e.g., *PKD1* + *COL4A5* co-inheritance), driving rapid progression.	Bilateral small cortical or medullary renal cysts mimicking early ADPKD, accompanied by a normal or small-sized total kidney volume.	High-depth NGS panel covering basement membrane and Alport syndrome loci.	[51,52,53,73]
*FLCN*	Monoallelic loss-of-function pathogenic variants in folliculin.	AD; phenocopy	Young adulthood.	Pediatric: Completely asymptomatic. Adult: Pulmonary, cutaneous, and renal neoplastic developments.	Variable, asymmetric renal cysts that can macroscopically mimic or phenocopy classical ADPKD.	Birt–Hogg–Dubé syndrome: associated with spontaneous pneumothorax, fibrofolliculomas, and elevated risk of renal tumors.	Variable, asymmetric renal macrocysts that structurally mimic or phenocopy classical adult ADPKD patterns.	Targeted gene sequencing or standard NGS panels.	[58,59]
*OFD1*	Pathogenic variants in the X-linked *OFD1* gene	X-linked dominant	Infancy to early childhood; lethal in males.	Pediatric: Severe multi-organ syndromic malformations and early-onset renal macrocysts. Adult: Severe progressive cystic kidney failure.	Prominent PKD-like renal cystic phenotype characterized by bilateral macrocysts.	Oral-facial-digital syndrome type 1: cleft palate, facial dysmorphisms, cognitive impairment, and distinct digital anomalies (important clinical clues).	Prominent PKD-like renal phenotype characterized by early development of bilateral macrocysts and diffuse parenchymal distortion.	Multigene ciliary NGS panel or targeted X-chromosome sequencing.	[74,75]
ADTKD genes	Mutations in *UMOD, MUC1, REN*, or *SEC61A1*	AD; interstitial spectrum	Late childhood to adulthood.	Pediatric: Hyperuricemia, enuresis, gout (REN/UMOD). Adult: Slowly progressive tubulointerstitial nephritis and late-onset ESKD.	Small renal cysts accompanied by marked tubulointerstitial fibrosis, progressive GFR decline, and impaired urinary concentrating capacity.	Represents a hybrid ADPKD/ADTKD phenotype; underscores the absolute necessity of multigene panel sequencing for differential diagnosis.	Bilateral small or normal-sized kidneys, increased parenchymal echogenicity, and few, small corticomedullary cysts.	Targeted NGS panels for tubulointerstitial diseases, with specialized assays for *MUC1* (e.g., SMRT sequencing).	[65,66,67]

Note: Table 1 outlines the main genes known to contribute to both classic and atypical forms of ADPKD. Alongside *PKD1* and *PKD2*, which account for the vast majority of clinical cases, several additional loci (such as *GANAB, DNAJB11, ALG9, ALG8, IFT140, NEK8, COL4A3–A5, PKHD1, FLCN, OFD1*, and *HNF1B*) are associated with milder, unusual, or overlapping phenotypes. Abbreviations: AD, autosomal dominant; ADPKD, autosomal dominant polycystic kidney disease; ADPLD, autosomal dominant polycystic liver disease; ADTKD, autosomal dominant tubulointerstitial kidney disease; AR, autosomal recessive; ARPKD, autosomal recessive polycystic kidney disease; CDG, congenital disorder of glycosylation; CHF, congenital hepatic fibrosis; CKD, chronic kidney disease; CMA, chromosomal microarray; ER, endoplasmic reticulum; ESKD, end-stage kidney disease; FLCN, folliculin; GFR, glomerular filtration rate; HEK, hyperechogenic kidneys; LR-PCR, locus-specific long-range polymerase chain reaction; MLPA, multiplex ligation-dependent probe amplification; MODY5, maturity-onset diabetes of the young type 5; NGS, next-generation sequencing; SMRT, single-molecule real-time sequencing; TKV, total kidney volume; TRP, transient receptor potential; VEO-ADPKD, very-early-onset autosomal dominant polycystic kidney disease; WES, whole-exome sequencing; WGS, whole-genome sequencing.

**Table 2:** Diagnostic criteria, imaging findings, and prognostic stratification across the lifespan.

Age/Cohort	Key Criteria and Imaging Findings	Practical and Molecular Notes	References
Fetus/Prenatal	Bilateral enlarged, HEK; increased CMD supports ADPKD, whereas decreased or lost CMD favors ARPKD. Severe cases present with oligohydramnios and subsequent pulmonary hypoplasia *	HEK can be detected as early as the 13–16-week gestation scan, though more typically at 18–20 weeks; fetal prognosis is strictly dependent on persistent amniotic fluid volume **	* [5,67,89,90,91,92,93,94,95,96] ** [67,93,94,95,96]
0–18 months (VEO-ADPKD)	Markedly enlarged, palpable kidneys associated with at least one systemic sign: blood pressure > 95th percentile for age/sex/height, eGFR < 90 mL/min/1.73 m^2^, or persistent proteinuria *	Frequently triggered by complex genetic mechanisms, including biallelic hypomorphic PKD1 variants or synergistic digenic inheritance patterns **	* [69,70,132,133] ** [27,69,70,72,97]
18 months–15 years (EO-ADPKD)	Enlarged or palpable kidneys accompanied by at least one clinical sign: early-onset hypertension, reduced GFR, or persistent proteinuria *	Many children still exhibit very few cysts at this stage; renal ultrasound may be falsely negative in milder or atypical genotypes **.	* [132,133] ** [50,123,127,128]
Child at risk (Positive family history)	Even a single solitary cyst is highly suspicious; the detection of two or more cysts is traditionally considered diagnostic *	Simple benign cysts are exceptionally rare in childhood; a detailed parental family history and serial ultrasound monitoring are essential **	* [11,123,124,125] ** [10,11,124,125,126]
16–40 years (Positive family history)	MRI-based criteria: >10 renal cysts strongly support the diagnosis; <5 cysts makes the condition highly unlikely *	Positive predictive value (PPV) is 100% for both *PKD1* and *PKD2*; however, diagnostic sensitivity is notably lower for *PKD2* (70% vs. 94% for *PKD1*) **	* [94,128,129] ** [12,17,30,31,32,128]
Adult with typical phenotype (Mayo Class 1)	Application of the Mayo Imaging Classification (MIC) utilizing age- and htTKV kinetics for prognostic risk stratification *	Strictly limited to typical, symmetric, and diffuse cystic patterns; not validated or applicable for non-*PKD1/PKD2* genotypes or atypical (Class 2) imaging patterns **	* [50,129,130,131] ** [43,49,50]
Pediatric stratification (Risk assessment)	Application of the Leuven Imaging Classification, alongside total cyst tracking (>10 cysts per kidney), baseline htTKV kinetics, and early hypertension or proteinuria *	Serves as the premier validated childhood prognostic model designed to differentiate “fast” from “slow” progressive renal phenotypes early in life **	* [115,118,134] ** [115,134,135,136,137]
Hybrid phenotypes (ADPKD-ADTKD overlap)	Small cysts accompanied by marked tubulointerstitial fibrosis; progressive GFR decline manifests with a relatively small number of cysts and near-normal TKV *	Suggestive of complex underlying pathogenic variants in alternative loci (e.g., *DNAJB11, HNF1B, UMOD, SEC61A1*); necessitates the rapid deployment of extended genetic panels **	* [50,65,66] ** [10,58,59,65,66]

Note: Table 2 provides a comprehensive review of key multi-modal imaging findings, diagnostic milestones, and risk-stratification criteria for ADPKD from fetal life through adulthood. It highlights the clinical utility of ultrasound and MRI across different age cohorts, defines the clinical thresholds for very-early and early-onset disease, and underscores the limitations of applying adult-validated prognostic frameworks. Abbreviations: ADPKD, autosomal dominant polycystic kidney disease; ADTKD, autosomal dominant tubulointerstitial kidney disease; ARPKD, autosomal recessive polycystic kidney disease; CMD, corticomedullary differentiation; EO-ADPKD, early-onset autosomal dominant polycystic kidney disease; eGFR, estimated glomerular filtration rate; GFR, glomerular filtration rate; HEK, hyperechogenic kidneys; htTKV, height-adjusted total kidney volume; MRI, magnetic resonance imaging; PPV, positive predictive value; TKV, total kidney volume; VEO-ADPKD, very-early-onset autosomal dominant polycystic kidney disease. s to pediatric or atypical populations. * Indicates the corresponding references marked with a single asterisk in the References column; ** Indicates the corre-sponding references marked with a double asterisk in the References column.

**Table 3:** Critical overview of contemporary knowledge gaps and clinical controversies in pediatric ADPKD and overlapping ciliopathies.

Domain	Current Evidence	Clinical Controversies & Knowledge Gaps	References
Prognostic Models	Multi-parametric adult frameworks (MIC/Mayo, PROPKD) predict rapid progression based on macro-cysts and adult milestones.	Absolute lack of pediatric-specific risk-prediction software. Adult htTKV kinetics completely fail to capture risk in children under 15 years and in atypical minor loci (*IFT140*, *GANAB*, *DNAJB11*) characterized by high fibrosis but near-normal kidney volume.	[50,129,131,134,135,138,140]
Hemodynamic Monitoring	Severe cardiovascular damage (increased LVMI) can develop silently in children even with normal office blood pressure.	Methodological debate on TKV normalization (height vs. body surface area adjustments in growing children) and lack of consensus on specific pediatric LDL-cholesterol targets for early metabolic comorbidities.	[104,113,116,118,119,121,122,141]
Advanced Imaging	MRI offers gold-standard volumetric precision in monitoring cyst kinetics without radiation risks.	Logistical and safety bottlenecks in infants. Routine utility is heavily restricted by motion artifacts and the frequent necessity for general sedation, while CT is avoided due to cumulative ionizing radiation risks.	[128,129,130,131,132]
Genetic Screening	Early molecular testing allows precise genotype–phenotype correlations and guides targeted ABPM protocols from age 5.	Ethical and psychosocial dilemmas. Balancing the immediate clinical benefit of early complication screening against the heavy psychological burden, family distress, and potential future insurance discrimination in asymptomatic minors.	[10,11,123,124,125,126,127,128]
Therapeutic Options	Tolvaptan is the standard disease-modifying therapy validated to slow adult cystogenesis by reducing cAMP-dependent proliferation.	Severe pediatric therapeutic void. No approved disease-modifying drugs are labeled for children. Translation is blocked by a lack of long-term safety data on growth and the risk of severe aquaretis-induced dehydration in school-aged cohorts.	[10,140,142,143,144,145,146]
Alternative Interventions	Pravastatin and insulin-sensitivity pathways (HOMA-IR) are being explored in pediatric trials and preclinical models.	Uncoupled functional benefits. Pravastatin attenuates htTKV growth but fails to modify global GFR or microalbuminuria. The direct causal link between intrinsic insulin resistance and early cyst kinetics remains unconfirmed.	[10,80,81,147,148,149]
Advanced Biology	Preclinical platforms (CRISPR/Cas9, RNA-modulators, mTOR/tyrosine kinase inhibitors) target primary ciliary defects.	Undetermined safety and delivery profiles. High risk of catastrophic off-target genomic effects in a growing child and lack of targeted vehicle delivery options to the renal parenchyma.	[38,56,85,140,150,151,152,153]
Complication Management	Tissue-invasive cyst infections require prolonged treatments (4–6 weeks) with highly lipid-soluble antimicrobial agents.	Pediatric toxicological paradox. First-line lipid-soluble drugs (e.g., fluoroquinolones) demand extreme clinical caution in youth due to documented musculoskeletal and cartilage toxicity.	[10,105,106,107]

Abbreviations: ABPM, ambulatory blood pressure monitoring; ADPKD, autosomal dominant polycystic kidney disease; cAMP, cyclic adenosine monophosphate; CRISPR/Cas9, clustered regularly interspaced short palindromic repeats/CRISPR-associated protein 9; CT, computed tomography; HOMA-IR, homeostatic model assessment for insulin resistance; htTKV, height-adjusted total kidney volume; LDL, low-density lipoprotein; LVMI, left ventricular mass index; MIC, Mayo Imaging Classification; mTOR, mechanistic target of rapamycin; MRI, magnetic resonance imaging; RNA, ribonucleic acid; TKV, total kidney volume.

**Table 4:** Current pharmacotherapy, experimental strategies, and supportive interventions for autosomal dominant polycystic kidney disease.

Drug/Intervention	Target/Molecular Mechanism	Evidence	Limitations	Pediatric Protocol (Dose and Duration)	References
Tolvaptan	Same mechanism (V2R signaling pathway).	Recent Phase 3 pediatric trial: showed a positive signal for curbing progressive kidney enlargement and preserving long-term eGFR.	Not yet approved in children; larger clinical cohorts and long-term safety/developmental data are needed before routine implementation.	Dose: Twice-daily split-dose regimen (morning/afternoon 8 h later) using 7.5, 15, or 30 mg tablets. Initial dose is weight-based: 15 mg/day for <40 kg; 30 mg/day for 40–60 kg; 45 mg/day for >60 kg. Up-titrated weekly based on tolerability to a maximum target of 90 mg/day (60 + 30 mg). Each dose must be taken with 240 mL of water. Duration: 12 months (1 year)	[140,145,146]
Vasopressin analogues (e.g., desmopressin)	V2R agonist; triggers systemic cAMP accumulation.	Contraindicated; actively promotes cyst proliferation and accelerates fluid secretion in vivo.	Not recommended in children diagnosed with or at high risk for ADPKD due to potential pro-cyst effects.	Contraindicated (Avoid use).	[10,11]
ACE inhibitors/ARBs	Systemic RAAS blockade → ↓ blood pressure, ↓ proteinuria.	First-line for hypertension and proteinuria in ADPKD; may slow long-term disease progression.	Mandatory in VEO/EO ADPKD cohorts; aggressive blood pressure targets are established at ≤50th percentile or ≤110/70 mmHg in older adolescents.	Dose: Individually titrated to achieve strict BP targets (<50th percentile). Commonly utilized agents include Enalapril (initiated at 0.08 mg/kg once daily up to a maximum of 10–40 mg/day) or Lisinopril (initiated at 0.07 mg/kg once daily up to a maximum of 5–40 mg/day based on age/weight tiers), titrated every 2–4 weeks. Duration: Chronic, lifelong management starting immediately upon confirmation of pediatric hypertension (at or above age 5 years)	[10,113,122,141]
Pravastatin	Pleiotropic endothelial and structural tissue effects.	Significantly reduced the rate of height-adjusted TKV (htTKV) increase in children; no clear benefit demonstrated on left ventricular mass index, microalbuminuria, or global GFR.	Potential structural-only parenchymal remodeling benefit; does not currently represent a standard of care.	Dose: 20 to 40 mg orally once daily. Specifically administered as a fixed dose of 20 mg/day orally for children aged 8 to 13 years, and 40 mg/day orally for adolescents aged 14 to 18 years, taken in the evening. Duration: 3 years (36 months).	[147,148]
Metabolic control (Obesity, insulin resistance)	Modulation of glucose-dependent signaling pathways.	Systemic insulin resistance and altered glucose metabolism are robustly associated with more rapid disease progression and accelerated cystogenesis.	Early lifestyle adjustments, obesity management, and monitoring of insulin sensitivity may protect long-term renal and cardiovascular outcomes.	Dose: No pharmacological dose established. Requires customized non-pharmacological dietary adjustments (low-osmolar diet, limited refined sugar intake) combined with regular physical exercise (30–60 min/day) to prevent childhood obesity. Duration: Continuous	[77,80,81,149]
mTOR-targeted therapies (e.g., rapalogs)	Inhibition of overactive mTOR signaling driven by PC1/TSC1–TSC2 complex dysfunction.	Strong biological and mechanistic rationale; however, clinical trial results have been mixed and unsatisfactory so far.	Limited by significant systemic toxicities, including immunosuppression, stomatitis, and dyslipidemia; not recommended for routine isolated ADPKD management.	Dose: Not recommended for routine pediatric use. Protocols are entirely unestablished in children with isolated ADPKD due to high systemic toxicity. Duration: None	[38,56,150]
Other agents (e.g., tepotinib, pasireotide)	MET receptor tyrosine kinase inhibitor, somatostatin analogue.	Currently under active clinical investigation for rapidly progressive ADPKD phenotypes and *TSC2/PKD1* contiguous gene syndrome (CGS).	Strictly experimental at this stage; not recommended or validated for pediatric cohorts.	Dose: Unapproved for general pediatric use; clinical doses are currently unestablished in children and restricted to adult-only trial designs. Duration: None.	[56,140,150,151]
Gene/RNA-based therapies	Editing, silencing, or modulating specific gene expressions.	High theoretical and curative potential in monogenic diseases using tools like CRISPR/Cas9 systems.	Early preclinical stages; substantial technical challenges remain regarding targeted kidney delivery, off-target mutations, and prohibitive economic costs.	Dose: Preclinical stages only Duration: No clinical pediatric protocol established	[152,153]

Note: Table 4 provides a comprehensive synthesis of current guideline-directed treatments, investigational disease-modifying agents, and non-pharmacological supportive interventions for autosomal dominant polycystic kidney disease. The table outlines targets within the vasopressin (V2R), metabolic, and mTOR signaling pathways, and highlights key clinical constraints, toxicities, and future biological avenues (such as gene editing and RNA modulation) aimed at advancing precision medicine in ADPKD management. Abbreviations: ACE, angiotensin-converting enzyme; ADPKD, autosomal dominant polycystic kidney disease; ARB, angiotensin II receptor blocker; BP, blood pressure; cAMP, cyclic adenosine monophosphate; CGS, contiguous gene syndrome; CRISPR/Cas9, clustered regularly interspaced short palindromic repeats/CRISPR-associated protein 9; eGFR, estimated glomerular filtration rate; EO, early-onset; GFR, glomerular filtration rate; htTKV, height-adjusted total kidney volume; MET, mesenchymal-epithelial transition factor; mTOR, mechanistic target of rapamycin; RAAS, renin-angiotensin-aldosterone system; RNA, ribonucleic acid; TSC, tuberous sclerosis complex; V2R, vasopressin V2 receptor; VEO, very-early-onset. The rightward arrow (→) indicates a causal transition or progression, and the downward arrow (↓) indicates a de-crease or reduction.

## Data Availability

No new data were created or analyzed in this study. Data sharing is not applicable to this article.

## Abbreviations

ABPM	ambulatory blood pressure monitoring
ACE	angiotensin-converting enzyme
AD	autosomal dominant
ADH	anti-diuretic hormone
ADPKD	autosomal dominant polycystic kidney disease
ADPLD	autosomal dominant polycystic liver disease
ADTKD	autosomal dominant tubulointerstitial kidney disease
ALG5	dolichyl-phosphate beta-glucosyltransferase gene
ALG6	dolichyl-phosphate alpha-glucosyltransferase gene
ALG8	alpha-1,3-glucosyltransferase gene
ALG9	alpha-1,2-mannosyltransferase gene
AR	autosomal recessive
ARPKD	autosomal recessive polycystic kidney disease
BBS	Bardet–Biedl syndrome
BMI	body mass index
BP	blood pressure
cAMP	cyclic adenosine monophosphate
CDG	congenital disorder of glycosylation
CGS	contiguous gene syndrome
CHF	congenital hepatic fibrosis
CKD	chronic kidney disease
CMA	chromosomal microarray
CMD	corticomedullary differentiation
COL4A3	collagen type IV alpha 3 chain gene
COL4A4	collagen type IV alpha 4 chain gene
COL4A5	collagen type IV alpha 5 chain gene
CRISP	Consortium for Radiologic Imaging Studies of Polycystic Kidney Disease
CRISPR/Cas9	clustered regularly interspaced short palindromic repeats/CRISPR-associated protein 9
CT	computed tomography
DNAJB11	DnaJ heat shock protein family member B11 gene
DOI	digital object identifier
DZIP1L	DAZ interacting zinc finger protein 1 like gene
eGFR	estimated glomerular filtration rate
EMA	European Medicines Agency
EO-ADPKD	early-onset autosomal dominant polycystic kidney disease
ER	endoplasmic reticulum
ESKD	end-stage kidney disease
ERK	extracellular signal-regulated kinase
FDA	Food and Drug Administration
FLCN	folliculin gene
G4	Guanine-quadruplex
GANAB	glucosidase alpha neutral AB gene
GFR	glomerular filtration rate
HEK	hyperechogenic kidneys
HNF1B	hepatocyte nuclear factor 1-beta gene
HOMA-IR	homeostatic model assessment for insulin resistance
HTD	hereditary tubulointerstitial disease
htTKV	height-adjusted total kidney volume
ICA	intracranial aneurysm
IFT140	intraflagellar transport 140
KDIGO	Kidney Disease: Improving Global Outcomes
LDL	low-density lipoprotein
LIC	Leuven Imaging Classification
LR-PCR	locus-specific long-range polymerase chain reaction
LVMI	left ventricular mass index
MAPK	mitogen-activated protein kinase
MCP-1	monocyte chemoattractant protein-1
MET	mesenchymal-epithelial transition factor
MIC	Mayo Imaging Classification
MLPA	multiplex ligation-dependent probe amplification
MODY5	maturity-onset diabetes of the young type 5
mRNA	messenger ribonucleic acid
mTOR	mechanistic target of rapamycin
NEK8	NIMA-related kinase 8 gene
NGAL	neutrophil gelatinase-associated lipocalin
NGS	next-generation sequencing
NPHP	nephronophthisis gene family
OFD1	oral-facial-digital syndrome type 1 gene
PCP	planar cell polarity
PKD	polycystic kidney disease
PKD1	polycystic kidney disease 1 gene
PKD2	polycystic kidney disease 2 gene
PKHD1	polycystic kidney and hepatic disease 1 gene
PROPKD	Predicting Renal Outcomes in Polycystic Kidney Disease score
RAAS	renin–angiotensin–aldosterone system
RASi	renin-angiotensin system inhibitors
SAH	subarachnoid hemorrhage
SMRT	single-molecule real-time sequencing
SONG-PKD	Standardised Outcomes in Nephrology-Polycystic Kidney Disease
TKV	total kidney volume
TMP-SMX	trimethoprim–sulfamethoxazole
TRP	transient receptor potential
TSC	tuberous sclerosis complex
TSC1	tuberous sclerosis complex gene 1
TSC2	tuberous sclerosis complex gene 2
US	ultrasound
UTI	urinary tract infection
V2R	vasopressin V2 receptor
VEO-ADPKD	very-early-onset autosomal dominant polycystic kidney disease
VUS	variant of uncertain significance
WES	whole-exome sequencing
WGS	whole-genome sequencing
Wnt	Wingless-related integration site
